# Radiographic evaluation of percutaneous transfacial wiring versus open internal fixation for surgical treatment of unstable zygomatic bone fractures

**DOI:** 10.1371/journal.pone.0220913

**Published:** 2019-08-15

**Authors:** Guillaume Giran, Arnaud Paré, Benjamin Croisé, Carine Koudougou, Jacques Marie Mercier, Boris Laure, Pierre Corre, Hélios Bertin

**Affiliations:** 1 Service de Chirurgie Maxillo-faciale et Stomatologie, Centre Hospitalier Universitaire de Nantes, Nantes, France; 2 Service de Chirurgie Maxillo-faciale et Plastique, Centre Hospitalier Universitaire de Tours, Chambray-lès-Tours, France; 3 Laboratoire de Médecine Régénératrice et Squelette (RMeS), Faculté de Chirurgie Dentaire, Nantes, France; 4 Laboratoire des Sarcomes Osseux et Remodelage des Tissus Calcifiés (Phy.Os), Faculté de Médecine, Nantes, France; Duke University School of Medicine, UNITED STATES

## Abstract

**Introduction:**

The fixation of unstable zygomaticomaxillary complex (ZMC) fractures can be achieved by open reduction with rigid internal fixation (ORIF) and/or by closed reduction with percutaneous transfacial Kirschner wire fixation (CRWF). The aim of this study was to tomographically assess the symmetry and the protrusion of the cheekbone with unstable ZMC fractures that had been treated by ORIF vs. CRWF.

**Materials and methods:**

Sixty patients exhibiting a surgically unstable tetrapodal ZMC fracture were included in this multicenter retrospective study. The coordinates of 5 landmarks representing the zygomatic protrusion were comparatively studied on the healthy and on the broken side using preoperative and postoperative tridimensional computed tomography (CT) scans or cone beam CT.

**Results:**

No significant difference was found in the zygomatic protrusion irrespective of the surgical technique that was used. The zygomatico-maxillary ansa was found to be the most complicated area to reduce, particularly in the frontal plane with both the CRWF and the ORIF technique (p_1_ = 0.001 and p_2_ = 0.0009, respectively). There was no difference in terms of the level of complications, while the mean duration of the surgery was significantly less for the CRWF group.

**Conclusion:**

With good postoperative radiographic outcomes, the CRWF can be proposed as an alternative or in association with the ORIF technique for fixation of tetrapodal fractures of the ZMC.

## Introduction

Fractures of the zygomaticomaxillary complex (ZMC) are a type of injury that is commonly encountered in maxillofacial surgery, accounting for 24% of all facial fractures [1,2]. Physical assaults, falls, traffic accidents, and sports-related injuries represent the most common etiologies, accounting for 39%, 31%, 11%, and 11% of all ZMC fractures, respectively [2]. Males outnumber females in most studies, with a sex ratio of 3.6/1. The mean age of the victims is between 30 and 40 years [2].

Computed tomography (CT) scan and cone beam CT (CBCT) are commonly used for imaged-based preoperative and postoperative evaluation of ZMC fractures. CT imaging with multiplanar and tridimensional (3D) reconstructions allows ZMC fractures to be defined [3]. As it is equally suitable for the examination of bones and as it involves less radiation, CBCT is replacing CT scans, with the exception of fractures of the orbital floor or when there is an associated brain injury [4].

The zygomatic bone is considered to be a tetrapod in which the four pillars can be broken (i.e., the zygomatic process of the frontal bone, the zygomatic process of the maxillary bone, the orbital rim, and the zygomatico-malar ansa). The treatment of such fracture aims to restore the normal anatomy of these pillars so as to improve the projection of the cheek and the symmetrical shape of the orbit. Furthermore, the sphenozygomatic suture represents an important site to be reduced, particularly in comminuted high-energy fractures [5]. A conservative treatment with simple reduction using a transcutaneous hook (Ginestet) is commonly used for ZMC fractures with minimal displacement. Surgical fixation is indicated for ZMC fractures that involve a significant degree of dislocation [6,7]. Open reduction with rigid internal fixation (ORIF) represents the standard of care to repair unstable zygomatic fractures [8–11] as the bone exposure allows for direct evaluation of the reduction. This technique necessitates various intraoral and/or cutaneous approaches (infraorbital, latero-orbital, coronal) depending on the number and the degree of dislocation [12,13]. There is currently a trend to hide the facial incisions (transconjunctival approach, upper eyelid incision) [14,15]. The osteosynthesis can be carried out by using a wire ligature, screw, or titanium or resorbable miniplate [16]. Because it represents a fast and mini-invasive approach, closed reduction with percutaneous transfacial Kirschner wire fixation (CRWF) can be proposed as an alternative for zygomatic fixation [16].

There are very few reports to date in the literature regarding the treatment of ZMC fractures using CRWF. Depending on the surgeon’s experience, the team, and the type of fracture, these different methods for fixation can be used and sometimes combined as a customized treatment. Nevertheless, the reasons for choosing one or the other fixation method are not clear; to our knowledge, there has been no study to date comparing these two fixation methods. The aim of this study was to tomographically assess the symmetry and the projection of the cheekbone after unstable ZMC fractures treated either by ORIF or by CRWF.

## Materials and methods

### Data collection

Sixty patients presenting with a unilateral ZMC fracture at the Oral and Maxillofacial Surgery Department of Nantes University Hospital (Center 1) or at the Maxillofacial and Facial Plastic Surgery Department of Tours University Hospital (Center 2) between 2010 and 2017 were included in the study and analyzed retrospectively. All of the patients exhibited a surgically unstable tetrapodal fracture, defined as a type B fracture according to the classification of Zingg et al. [17]. Patients with a fracture of the contralateral ZMC, an isolated zygomatic arch fracture, a ZMC comminutive fracture, or any combined mid-face fracture were excluded from the study. Patients who did not have preoperative or postoperative CT images were also excluded. The medical charts were reviewed and data documenting their date of birth, the side involved, the mechanism of the fracture, clinical findings, the type of surgery, and the duration of the surgery were compiled.

In this retrospective study, no change to the current clinical practice or randomization was performed. Due to the retrospective nature of this study, it was granted a written exemption from approval by the ethics committee of the Nantes University Hospital, according to Articles L. 1121–1 paragraph 1 and R 1121–2, paragraph 1 of the French Public Health Code.

### Surgical technique

Irrespective of the surgical approach used, a close reduction with a Ginestet hook was carried out. Fixation was performed when necessary in case of unstable fractures. The stability was checked manually by applying digital pressure.

**Fig 1:**
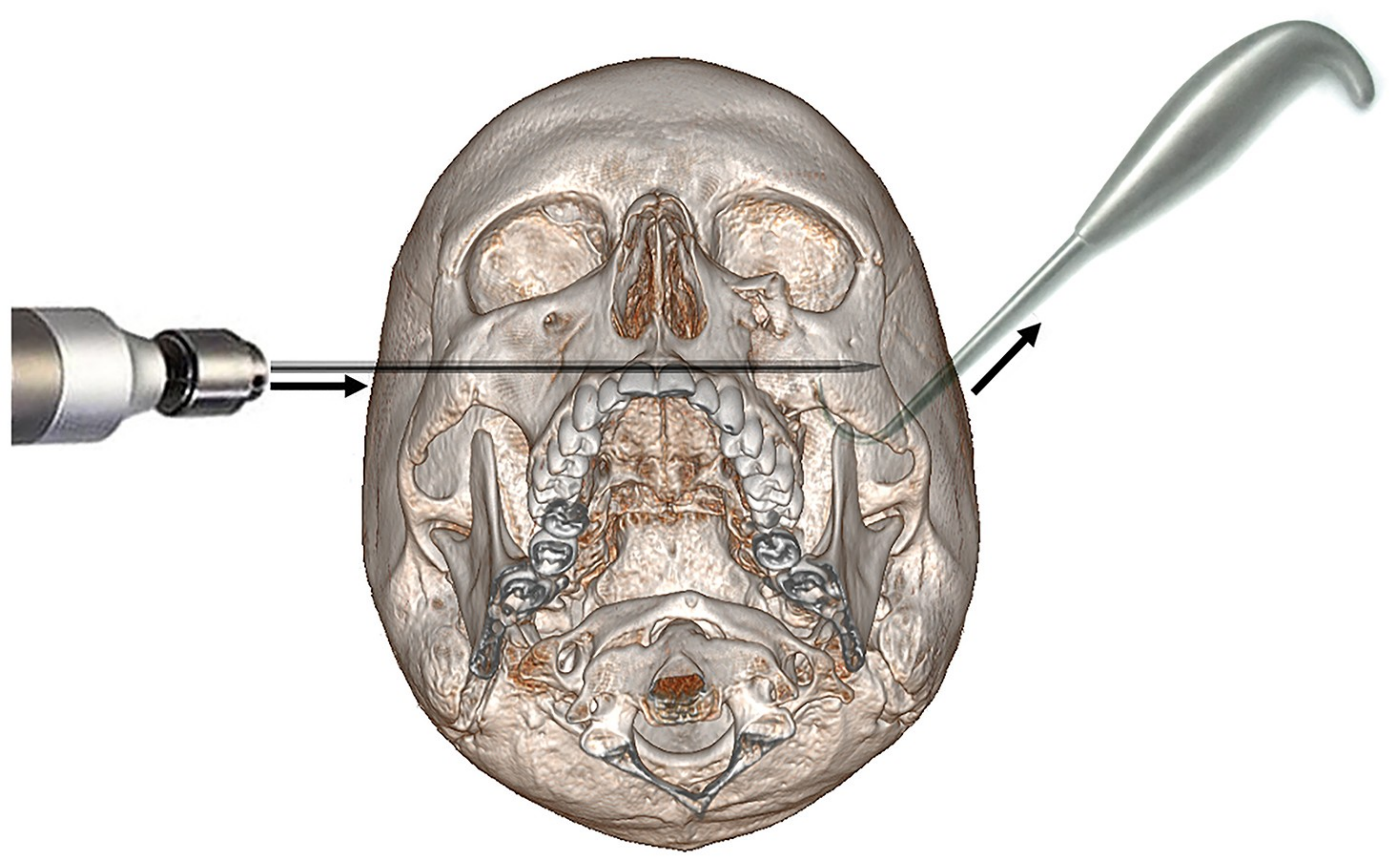
Representation of the CRWF technique. The broken zygomatic bone (left cheek) is maintained reduced with a Ginestet hook thanks to an upward pull, while the Kirschner wire is introduced into the corpus of the healthy side (right cheek) and pushed through the midfacial complex up to the broken zygomatic bone. The wire is then left for 3 weeks to allow bone consolidation.

The CRWF was provided at Center 1. Using a power drill, a Kirschner wire (0.071 inches or 1.8 mm in diameter) was introduced into the corpus of the healthy contralateral zygomatic bone, then passed through the maxillary sinus, the nasal septum, and pinned to the inner cortex of the broken zygomatic bone (Fig 1). In a few cases, a titanium wire loop was placed on the frontozygomatic suture using a palpebral approach.

The ORIF was performed at Center 2. Depending on the displacement, a subciliary, transconjunctival, eyebrow/palpebral, or intraoral approach was performed to expose the infra-orbital rim as well as the frontozygomatic and zygomaticomaxillary sutures. When anatomic reduction was obtained, internal fixation was performed using various types of miniplates (e.g., a straight 5-hole plate for a frontozygomatic fracture, an L-shaped plate for a zygomaticomaxillary fracture, or an arciform plate for the inferior orbital rim).

### Tomographic evaluation

**Fig 2:**
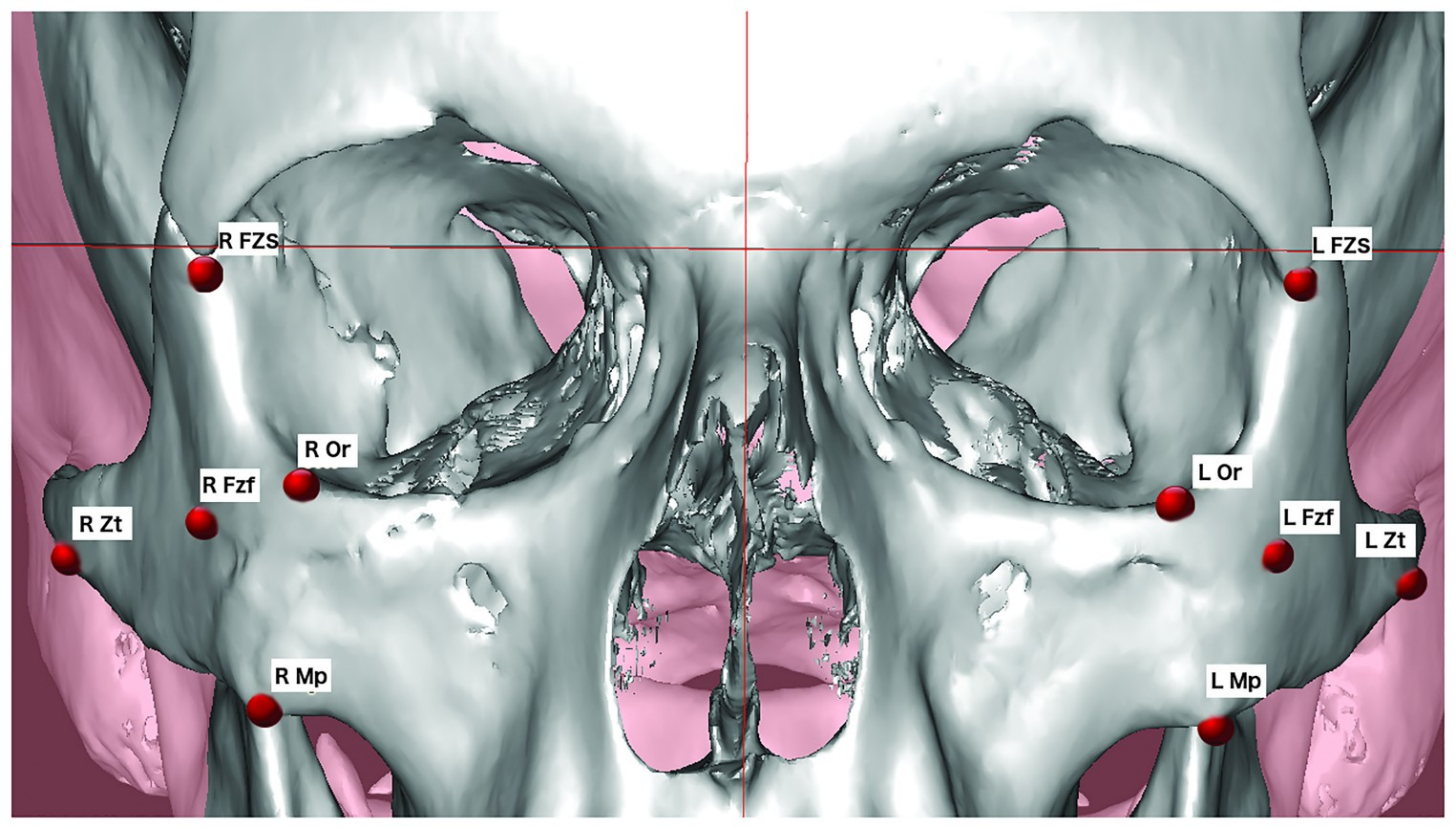
Representation of the five landmarks used for the cephalometric analysis of the zygomatic bone on 3D images. Fzf, foramen of the zygomaticofacial nerve; FZS, zygomaticofrontal suture; Mp, zygomaxillare point; Zt, zygotemporale inferior; Or, orbitale; R, right side; L, left side.

Preoperative and postoperative CT scans or CBCT were comparatively analyzed. Landmarks were placed manually by the same operator (other than the surgeons), either on sagittal, coronal, or axial sequences using Simplant O&O software (Materialise Dental N.V., Leuven, Belgium). Five landmarks were drawn on 3D images to assess the ZMC projection. The four classical landmarks Zygomaxillare (Mp), Orbitale (Or), Zygotemporale inferior (Zt), and Zygomaticofrontale (FZS) were chosen to represent each pillar of the zygomatic tetrapod (Fig 2). These anatomical points of the skull base were considered to be stable in light of their use in craniometry analysis, forensic sciences, and anthropology [18]. The fifth landmark, chosen as it is reproducible, was the foramen of the zygomaticofacial nerve (Fzf), which represents the projection of the zygomatic body [19]. These landmarks are listed in Table 1.

**Table 1:** The skeletal landmarks used for cephalometric measurements of the zygomatic bone position in 3D reconstructions of the CT scan and CBCT.

*Name*		*Definition*
Foramen of the zygomaticofacial nerve	Fzf	Opening of the zygomaticofacial branch of the trigeminal nerve at the center of the zygomatic bone
Zygomaxillare point	Mp	Lowest point of the zygomaticomaxillary suture
Zygotemporale inferior point	Zt	Lowest point of the zygomaticotemporal suture, at the top of the zygomatic tubercle
Zygomaticofrontale suture	FZS	Cranial suture between the zygomatic bone and the frontal bone
Orbitale	Or	Lowest point on the inferior edge of the orbit

**Fig 3:**
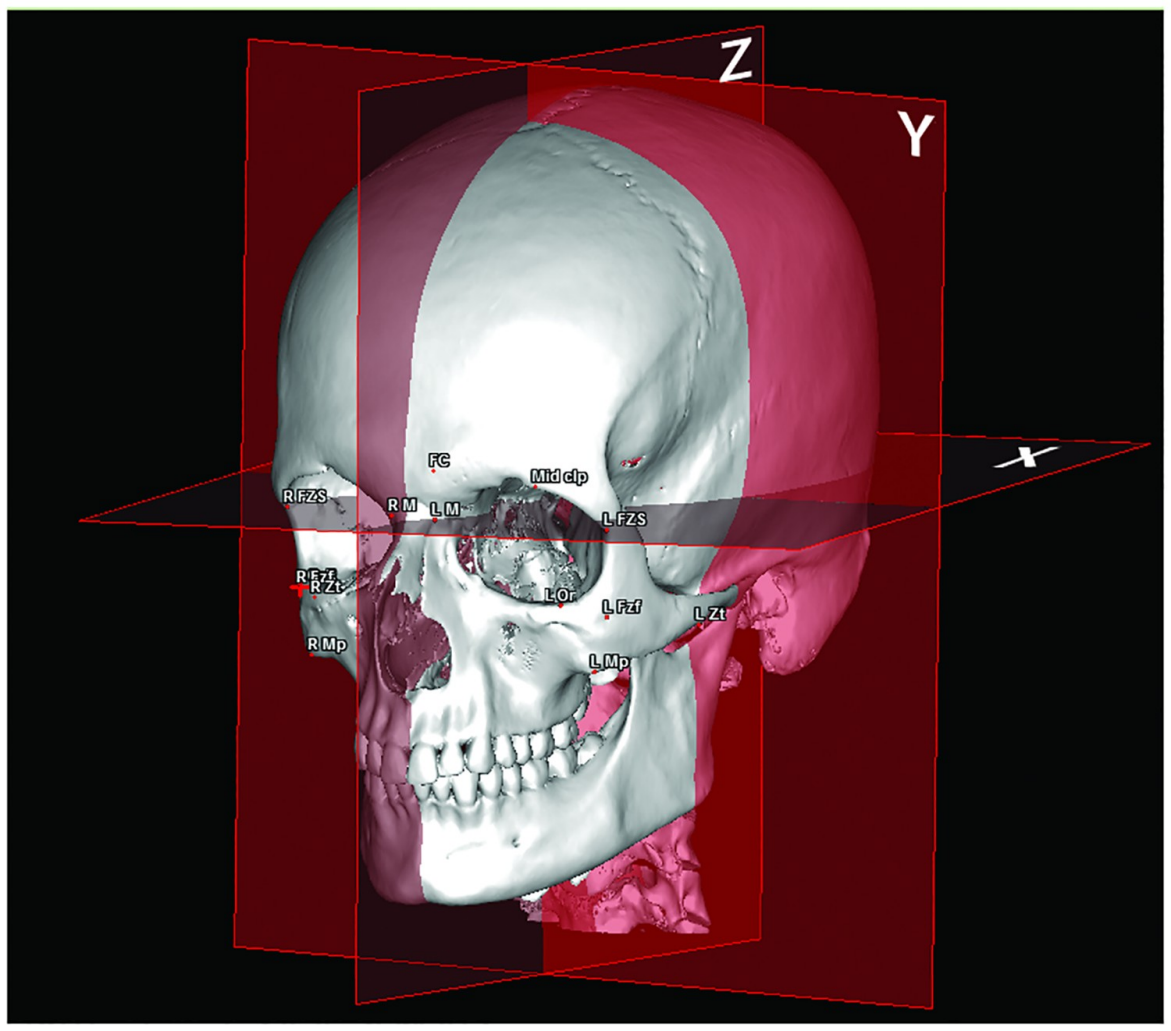
Orthonormal coordinate system constructed in the X, Y, and Z planes to determine the corresponding coordinates of the landmarks. The X-plane is the horizontal plane passing through the midpoint of the fronto-nasal suture (MidM) and of the posterior clinoid process (MidClp). The Y-plane is the vertical frontal plane passing through MidClp. The Z-plane corresponds to the vertical sagittal plane passing through MidClp, MidM, and the foramen caecum (Fc).

An orthonormal coordinate system was constructed as follows (Fig 3):

1. The Z median plane passing through the midpoint of the fronto-nasal suture (MidM), the midpoint of the posterior clinoid process (MidClp), and the foramen caecum (Fc).
2. The X-plane, corresponding to the 3D reconstruction of the C1 line of Delaire cephalometric analysis [20], perpendicular to the Z-plane, and passing through MidM and MidpClp.
3. The Y-plane, representing a 3D reconstruction of the C0 line described by Nimersken for the Delaire’s cephalometric analysis [21], constructed perpendicular to Z and X, and passing through MidClp.

The results obtained were the distance, expressed in mm, in an orthonormal basis with three-dimensional coordinates. Landmarks for the zygomatic projection were then studied comparatively between the broken side and the healthy side. The two fixation techniques were then compared in terms of the outcomes obtained in postoperative versus preoperative times.

### Statistical analysis

In 20 randomly selected patients, the cephalometric landmarks were positioned by a different operator in order to determine concordance. The methodical error of cephalometric measurements was assessed by Dahlberg’s formula (mean square error (S.E^2^) = d^2^/2N, where d is the difference between the first and the second measurements and N is the number of double measurements) [22].

The statistical analysis was performed using GraphPad Prism 6.0 software (GraphPad Software, La Jolla, CA, U.S.A.). The parametric function of our series was tested with a Shapiro-Wilk test. Comparative analysis of non-inferiority was carried out with a confidence interval of [-2.5; 2.5], considering that an asymmetry of 2.5 mm was the limit for an acceptable result [23]. The data were secondarily adjusted for age and gender. The quantitative data (i.e., comparison between the healthy and the broken side, and between the preoperative and the postoperative evaluation) were analyzed using a paired t-test for paired observations and a Mann-Whitney test for non-paired values. A p-value less than 0.05 (p<0.05) was taken to be statistically significant.

## Results

### Epidemiological data

Thirty patients presenting with a surgical ZMC fracture were reviewed and included in each center. Of the included patients, 48 (80%) were men and 12 (20%) were women. The average age at the time of the surgical procedure was 35.5 ± 16.4 years at Center 1 and 40.1 ± 18.9 years at Center 2 (Table 2). We noted a significant predominance of the affected side being on the left malar bone (60% versus 40% for the right side). According to the etiologies of the fractures, physical assaults represented 40% of the cases, followed by traffic accidents (22%), falls (20%), and sports-related injuries (18%).

**Table 2:** Patient characteristics.

	*Center 1 (N = 30)*	*Center 2 (N = 30)*	*TOTAL (N = 60)*
Females/Males, n (%)	7 (22%) / 23 (78%)	5 (17%) / 25 (83%)	12 (20%) /48 (80%)
Age (years), mean ± SD	35.5 ± 16.4	40.1 ± 18.9	37.8 ± 17.5
Right/left broken side, n (%)	16 (53%) / 14 (47%)	8 (27%) / 22 (73%)	24 (40%) /36 (60%)
Physical assault, n (%)	14 (47%)	10 (33%)	24 (40%)
Road traffic accident, n (%)	4 (13%)	9 (31%)	13 (22%)
Fall, n (%)	5 (17%)	7 (23%)	12 (20%)
Sports, n (%)	7 (23%)	4 (13%)	

n, number of patients; SD, standard deviation.

In regard to the clinical findings, 57 patients (95%) had a lack of zygomatic protrusion that was clinically objectivized. The three other patients had a substantial edema or no clinical issues. Fifty-nine patients (98%) presented with an infraorbital nerve (V2) sensitivity disorder. For fourteen patients (23%), their ability to open their mouth was limited. Twelve patients (20%) presented an associated fracture of the orbital floor, 5 in Center 1 and 7 in Center 2, treated by surgical transconjunctival approach. These patients exhibited a binocular diplopia in 5 cases (8%), while one case of oculomotor impairment was noted. Ten patients exhibited facial wounds, preferentially affecting the eyelid, the cheek, and the eyebrows.

In the ORIF group, for 13 patients (43%), only the frontozygomatic suture and orbital rim were approached, and osteosynthesis was carried out using one miniplate in each site. The 17 other patients (57%) needed a third fracture fixation of the zygomaticomaxillary buttress. In the CRWF group, for two patients, the Kirschner wire was associated with a titanium wire loop placed on the frontozygomatic suture using a palpebral approach.

### Primary outcome

The mean Dahlberg standard error for the cephalometric measurement was 0.81 mm ± 0.38 mm (0.23–1.64).

The projection of the broken side was compared between the ORIF and the CRWF groups in the preoperative period. No significant difference in the zygomatic coordinates was observed, thus suggesting that the extent of the displacement of the fracture was the same for the two groups.

The difference in the zygomatic protrusion between the healthy and the broken sides was comparatively studied in the two surgical groups (i.e., CRWF vs. ORIF). This revealed no significant difference, irrespective of the landmark that was studied (Table 3). The Mp point was found to fluctuate the most in the Y (i.e., the MpY point) and X (i.e., the MpX point) planes between the groups, albeit with no statistical significance (mean differences of 0.76 ± 0.83 mm and 0.60 ± 0.59 mm, respectively). The FzfY, ZFSY, and OrX landmarks also varied between the two surgical groups. Adjustments for age and gender revealed no significant differences.

**Table 3:** Non-inferiority test, comparison of the differences for the zygomatic protrusion between the healthy and the broken sides for the two fixation techniques.

*Differences*	*CRWF* *Mean (mm)*	*ORIF* *Mean (mm)*	*Difference* *CRWF—ORIF*	*95% CI*	*Interpretation*
ROrX-LOrX	0.99	1.60	-0.6087	[-1.1604; -0.0570]	Equivalent
ROrY-LOrY	2.19	1.73	0.4607	[-0.3341; 1.2554]	Equivalent
ROrZ-LOrZ	1.99	2.11	-0.1193	[-0.1193; -0.8829]	Equivalent
RZFSX-LZFSX	1.08	1.57	-0.4870	[-0.9795; 0.0055]	Equivalent
RZFSY-LZFSY	1.56	2.18	-0.6230	[-1.3249; 0.0789]	Equivalent
RZFSZ-LFZSZ	1.14	0.67	0.4767	[0.1096; 0.8437]	Equivalent
RFzfX-LFzfX	1.86	2.32	-0.4550	[-1.2693; 0.3593]	Equivalent
RFzfY-LFzfY	1.66	2.38	-0.7243	[-1.5652; 0.1165]	Equivalent
RFzfZ-LFzfZ	1.93	1.67	0.2563	[-0.4210; 0.9337]	Equivalent
RMpX-LMpX	1.27	1.87	-0.6013	[-1.1921; -0.0106]	Equivalent
RMpY-LMpY	2.53	1.77	0.7593	[-0.0759; 1.5945]	Equivalent
RMpZ-LMpZ	1.41	1.29	0.1260	[-0.6244; 0.8764]	Equivalent
RZtX-LZtX	2.08	2.27	-0.1960	[-1.0405; 0.6485]	Equivalent
RZtY-LZtY	2.46	2.31	0.1443	[-0.8892; 1.1779]	Equivalent
RZtZ-LZtZ	2.07	1.93	0.1433	[-0.5970; 0.8837]	Equivalent

Confidence Interval: [-2.5; 2.5]. For each landmark studied, R corresponds to the right side, L corresponds to the left side, X/Y/Z correlates with the dimensional coordinates. Or, orbitale point; ZFS, zygomaticofrontale suture landmark; Fzf, foramen of the zygomaticofacial nerve; Mp, zygomaxillare point; and Zt, zygotemporale inferior point.

**Fig 4:**
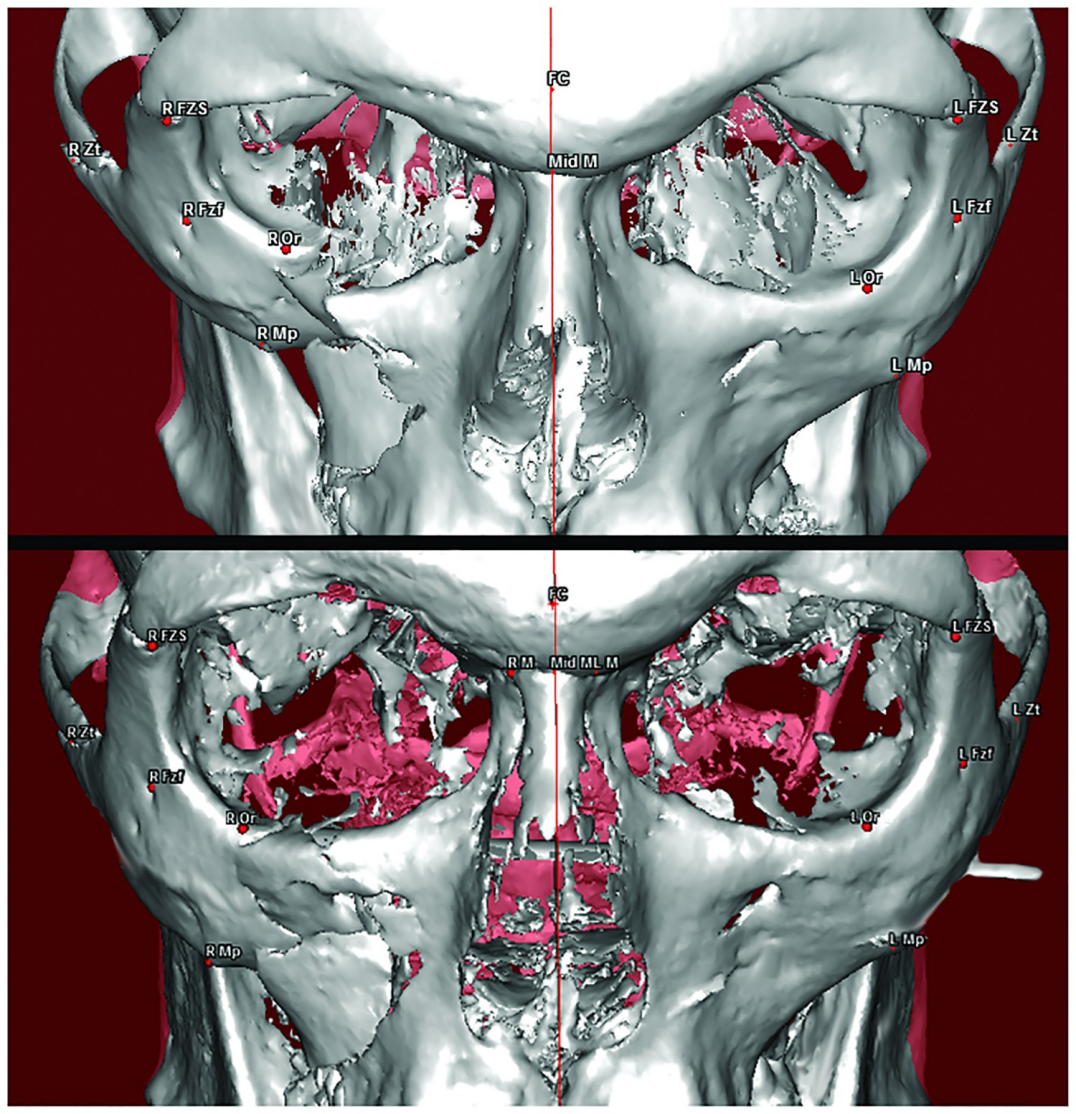
Three-dimensional reconstruction of the CT scan and the cone beam CT in a patient with a right tetrapodal ZMC fracture treated by the CRWF technique, preoperatively (top), and postoperatively (bottom) showing good reduction of the fracture.

In the patients treated with CRWF, the landmarks MpZ and MpY were significantly displaced postoperatively in the broken side compared to the healthy side, with mean values of 41.51 mm vs. 43.74 mm (p = 0.004) and 42.42 mm vs. 44.82 mm (p = 0.001), respectively (Fig 4).

**Fig 5:**
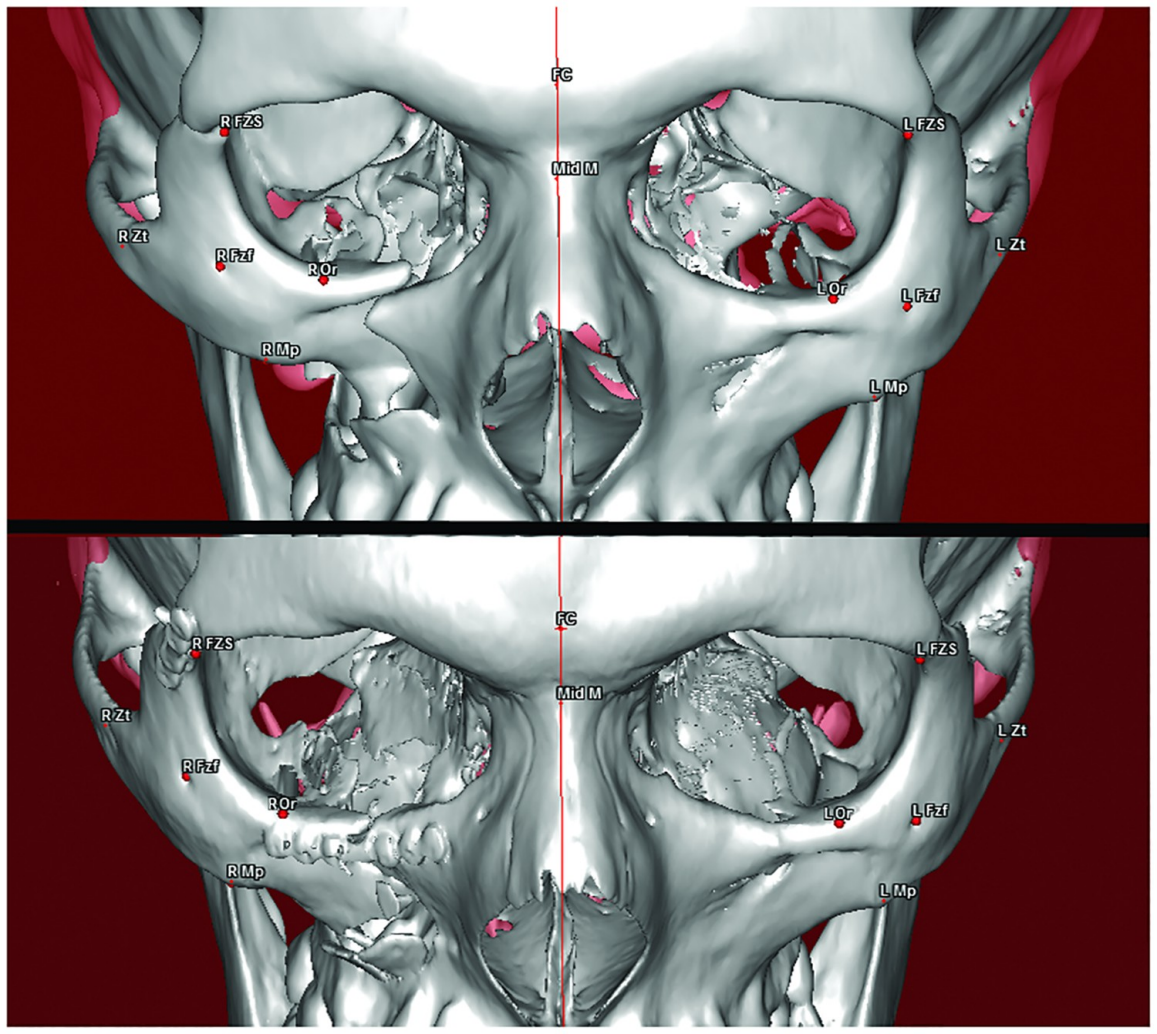
Three-dimensional reconstruction of the CT scan in a patient with a right tetrapodal ZMC fracture treated by the ORIF technique (lower orbital rim and fronto-zygomatic miniplates), preoperatively (top), and postoperatively (bottom) showing good radiographic outcomes in terms of the bone reduction.

In the patients treated with ORIF, a significant postoperative variation was found in the position of the MpY point in the broken side versus the healthy side (43.62 mm vs. 46.30 mm, p = 0.0009) (Fig 5).

### Secondary endpoints

The duration of the surgery was compared between the two groups, and it was found to be significantly shorter for the CRWF group than for the ORIF group, at 28.32 min (18–45) vs. 107.7 min (32–202), respectively (p<10^−4^).

The surgical complications did not differ between the two techniques. Acute maxillary sinusitis was observed in one patient of the CRWF group, and one infection on the maxillary buttress osteosynthesis was noted in a patient of the ORIF group. These infections were successfully treated using antibiotic therapy and osteosynthesis removal.

## Discussion

Surgical restoration of protrusion of the cheek can be a challenge with ZMC fractures. When contention is necessary, two surgical techniques can be proposed [16]. The ORIF method is presently the most commonly used technique [8]. The miniplates are widely available in most hospitals, and the current generation of facial surgeons has more experience with such procedures. The ORIF method allows open reduction of the different pillars of the zygomatic bone, but for some authors it results in facial scars when a subciliary/eyebrow approach is carried out [24,25]. Transconjunctival and the upper eyelid blepharoplasty incisions allow facial scars to be hidden in the surgical management of zygomatic fractures [14,15]. The Kirschner wire transmalar fixation technique is an old and well-known procedure [26–28]. It is still used by various teams, alone or in association with ORIF techniques, with good surgical outcomes [16]. However, with CRWF, removal of the K-wire under local anesthesia at least three weeks after implantation is systematically required. In terms of ORIF, although some authors argue for removal of the osteosynthesis devices, there is no real proof that this is of significant benefit in adults [29, 30].

Our study aimed to compare both of the ORIF and CRWF surgical techniques in terms of the zygomatic projection in patients exhibiting type B tetrapodal fractures of the ZMC. We found that, based on the postoperative tomographic assessments, the zygomatic position in the broken side was the same as in the healthy side, irrespective of the surgical technique that was used. The robustness of these findings is boosted by the fact that the preoperative images revealed the same severity of the fracture. These results are concordant with those obtained by Raoul *et al*. showing a high benefit/risk ratio for the patients in terms of the cost of the materials, the time to perform the surgery, and the degree of scarring in a series of 216 patients treated with CRWF [16]. The K-wire fixation can also be associated with osteosynthesis of the lateral orbital rim to provide a higher level of stability of the reduced zygomatic bone [31]. Other authors have stated that most surgeons find miniplate fixation easier to use than wire osteosynthesis, while more experience is required for K-wire fixation [32]. In our study, the CRWF technique was carried out by twelve different surgeons, most of who were junior surgeons with limited experience regarding the technique. This procedure is probably much more difficult to teach and to get experience with since the reduction and the repositioning of the bone fragment are not under visual control. The main clinical criterion to assess the quality of the reduction remains the symmetry of the broken side compared to the healthy side, as intraoperative cone beam CT is not used in the study centers.

Our findings underscore that the Mp landmark, representing the zygomatico-maxillary ansa, was the most complicated area to reduce, particularly with the CRWF technique. This insufficient reduction can be explained by the occurrence of a comminuted fracture, and hence difficulty with controlling the reduction in this area. The zygomatico-maxillary ansa is in fact anatomically hidden under a soft component (the malar fat pad, the zygomaticus muscles, and the masseter) and does not significantly affect the esthetic and functional outcomes.

By using X-, Y-, and Z-planes with the usual anatomical cephalometric landmarks, the model used in this study is readily reproducible. The four landmarks exploited to represent the zygomatic shape are commonly used in anthropometry and anthropology sciences [18]. However, the zygomaticofacial foramen has rarely been described in the literature. Although it can vary anatomically between individuals, it remains easy to process when analyzed in the same patient [19]. The low Dahlberg score found in our work suggests a high reproducibility of the landmark evaluation method. The advent of surface CBCT will help maxillofacial surgeons to measure the projection of the soft parts more objectively, in addition to enhancing the esthetic results of the surgery [33].

No difference was observed between the two techniques in terms of surgical complications, while a reduced operative time was noted with the CRWF technique. The advantages/disadvantages of CRWF and ORIF are summarized in Table 4.

**Table 4:** Comparison of the advantages and disadvantages expected with the ORIF and CRWF surgical techniques.

*Surgical technique*	*Advantages*	*Disadvantages*
CRWF	Reduced operative time	Removal of the wire required under local anesthesia
	Minimally invasive approach	No visual control of the bone reduction
	No materials remain after wire removal	No approach of the orbital floor
	Low price of the device	
ORIF	Visual control of the bone reduction	Increased operative time
	Approach of the orbital floor	Facial scar (subciliary approach)
		Materials remain
		Increased price of the device

CRWF, closed reduction with wire fixation; ORIF, open reduction with internal fixation.

Our study suffers from some limitations: the first one is that the long-term stability was not comparatively studied for the two surgical techniques. Furthermore, it was based on retrospective data, and the surgical procedures were performed at two different units, by numerous surgeons.

Our study calls for prospective and randomized controlled studies that include a larger sample population to compare osseous and soft tissue projection results for both of the CRWF and ORIF techniques in ZMC fractures.

## Conclusion

ZMC fractures are very common in maxillofacial surgery, and they usually require surgical treatment. With good postoperative radiographic outcomes and a shorter operative time, the CRWF can be proposed as an alternative or in association with the ORIF technique for the fixation of tetrapodal fractures of the ZMC.

## Supporting information

S1 Table Coordinates values, expressed in millimeters, for each patient (from 1 to 30) before the surgical procedure in Center 1 (CRWF group). For each landmark studied, R corresponds to the right side, L corresponds to the left side, X/Y/Z correlates with the three-dimensional coordinates (X, axial plane; Y, coronal plane; Z, sagittal plane). Or, orbitale landmark; ZFS, zygomaticofrontale suture landmark; Fzf, foramen of the zygomaticofacial nerve; Mp, zygomaxillare point; Zt, zygotemporale inferior point.
(DOC)

S2 Table Coordinates values, expressed in millimeters, for each patient (from 1 to 30) before the surgical procedure in Center 2 (ORIF group). For each landmark studied, R corresponds to the right side, L corresponds to the left side, X/Y/Z correlates with the three-dimensional coordinates (X, axial plane; Y, coronal plane; Z, sagittal plane). Or, orbitale landmark; ZFS, zygomaticofrontale suture landmark; Fzf, foramen of the zygomaticofacial nerve; Mp, zygomaxillare point; Zt, zygotemporale inferior point.
(DOC)

S3 Table Coordinates values, expressed in millimeters, for each patient (from 1 to 30) after the surgical procedure in Center 1 (CRWF group). For each landmark studied, R corresponds to the right side, L corresponds to the left side, X/Y/Z correlates with the three-dimensional coordinates (X, axial plane; Y, coronal plane; Z, sagittal plane). Or, orbitale landmark; ZFS, zygomaticofrontale suture landmark; Fzf, foramen of the zygomaticofacial nerve; Mp, zygomaxillare point; Zt, zygotemporale inferior point.
(DOC)

S4 Table Coordinates values, expressed in millimeters, for each patient (from 1 to 30) after the surgical procedure in Center 2 (ORIF group). For each landmark studied, R corresponds to the right side, L corresponds to the left side, X/Y/Z correlates with the three-dimensional coordinates (X, axial plane; Y, coronal plane; Z, sagittal plane). Or, orbitale landmark; ZFS, zygomaticofrontale suture landmark; Fzf, foramen of the zygomaticofacial nerve; Mp, zygomaxillare point; Zt, zygotemporale inferior point.
(DOC)
