# Supplementary material for: Radiographic evaluation of percutaneous transfacial wiring versus open internal fixation for surgical treatment of unstable zygomatic bone fractures
Source: PLoS One. 2019 Aug 15;14(8):e0220913. doi: 10.1371/journal.pone.0220913 (PMC6695106; doi:10.1371/journal.pone.0220913)
Supplement: S1 Table — For each landmark studied, R corresponds to the right side, L corresponds to the left side, X/Y/Z correlates with the three-dimensional coordinates (X, axial plane; Y, coronal plane; Z, sagittal plane). Or, orbitale landmark; ZFS, zygomaticofrontale suture landmark; Fzf, foramen of the zygomaticofacial nerve; Mp, zygomaxillare point; Zt, zygotemporale inferior point. (DOC) [file pone.0220913.s001.doc]

S1 Table.

| ***Patient*** | ***RZFSZ*** | ***RZFSX*** | ***RFZSY*** | ***RZtZ*** | ***RZtY*** | ***RZtX*** | ***RMpZ*** | ***RMpY*** | ***RMpX*** | ***RFzfZ*** | ***RFzfY*** | ***RFzfX*** | ***ROrZ*** | ***ROrY*** | ***ROrX*** | ***LOrZ*** | ***LOrY*** | ***LOrX*** | ***LZtZ*** | ***LZtY*** | ***LZtX*** | ***LMpZ*** | ***LMpY*** | ***LMpX*** | ***LZFSZ*** | ***LZFSY*** | ***LZFSX*** | ***LFzfZ*** | ***LFzfY*** | ***LFzfX*** |
| --- | --- | --- | --- | --- | --- | --- | --- | --- | --- | --- | --- | --- | --- | --- | --- | --- | --- | --- | --- | --- | --- | --- | --- | --- | --- | --- | --- | --- | --- | --- |
| 1 | 47,55 | 2,39 | 54,61 | 64,12 | 22,26 | 32,71 | 41,26 | 37,02 | 45,75 | 47,55 | 44,99 | 26,36 | 31,70 | 49,28 | 24,18 | 36,30 | 59,10 | 27,00 | 62,77 | 19,04 | 31,56 | 46,30 | 42,88 | 49,99 | 48,93 | 56,65 | 2,48 | 48,59 | 50,36 | 31,99 |
| 2 | 46,95 | 3,50 | 52,44 | 54,61 | 27,98 | 38,14 | 45,15 | 41,34 | 46,75 | 46,95 | 45,34 | 24,1 | 33,18 | 57,51 | 30,66 | 33,31 | 60,79 | 29,32 | 58,22 | 25,96 | 30,33 | 44,09 | 44,69 | 45,89 | 46,77 | 54,96 | 2,38 | 49,25 | 48,28 | 20,68 |
| 3 | 48,98 | 0,85 | 50,52 | 60,42 | 23,78 | 30,97 | 42,61 | 44,90 | 41,77 | 48,98 | 44,39 | 20,49 | 39,42 | 52,41 | 23,23 | 39,08 | 56,46 | 23,27 | 56,40 | 25,80 | 29,62 | 44,37 | 48,18 | 41,08 | 49,35 | 53,36 | 0,49 | 47,32 | 49,47 | 25,23 |
| 4 | 49,77 | 3,48 | 55,22 | 59,43 | 25,22 | 32,88 | 43,95 | 45,24 | 46,94 | 49,77 | 52,31 | 30,22 | 35,00 | 62,75 | 28,46 | 32,37 | 53,86 | 26,19 | 59,86 | 19,85 | 29,42 | 37,84 | 37,79 | 43,96 | 47,68 | 53,86 | 0,82 | 40,89 | 49,11 | 29,32 |
| 5 | 53,00 | 0,76 | 51,67 | 63,86 | 18,85 | 35,19 | 37,37 | 34,00 | 43,21 | 53,00 | 42,23 | 27,28 | 36,16 | 45,53 | 24,17 | 36,05 | 49,63 | 24,86 | 60,85 | 16,22 | 31,28 | 40,86 | 36,81 | 45,71 | 51,62 | 49,85 | 1,25 | 49,62 | 42,87 | 24,24 |
| 6 | 49,67 | 2,06 | 57,76 | 54,65 | 28,00 | 31,92 | 42,85 | 54,27 | 46,77 | 49,67 | 53,71 | 26,90 | 38,47 | 69,27 | 27,77 | 38,33 | 64,57 | 27,19 | 57,1 | 30,27 | 33,19 | 42,29 | 52,81 | 45,20 | 48,73 | 59,19 | 0,02 | 49,84 | 53,22 | 27,79 |
| 7 | 43,79 | 4,53 | 45,21 | 49,62 | 15,51 | 30,76 | 33,76 | 34,29 | 45,26 | 43,79 | 41,76 | 27,68 | 29,8 | 49,83 | 23,28 | 32,21 | 51,98 | 22,12 | 52,98 | 16,24 | 28,62 | 40,81 | 37,80 | 43,51 | 44,27 | 44,98 | 2,95 | 43,65 | 42,06 | 26,04 |
| 8 | 50,20 | 1,97 | 48,41 | 66,79 | 17,08 | 32,14 | 46,79 | 32,09 | 46,63 | 50,20 | 37,89 | 26,36 | 37,56 | 44,76 | 27,28 | 35,73 | 53,64 | 27,72 | 63,99 | 21,78 | 32,39 | 47,42 | 39,92 | 46,43 | 51,39 | 50,39 | 2,68 | 51,18 | 45,37 | 27,10 |
| 9 | 50,71 | 4,61 | 53,53 | 64,67 | 20,84 | 33,03 | 39,19 | 39,30 | 50,27 | 50,71 | 42,25 | 32,21 | 37,68 | 49,61 | 26,30 | 39,32 | 58,09 | 29,04 | 62,12 | 20,43 | 31,62 | 45,58 | 45,97 | 51,73 | 54,83 | 55,36 | 3,80 | 54,62 | 46,70 | 34,00 |
| 10 | 46,27 | 2,06 | 50,33 | 56,94 | 20,64 | 31,12 | 34,79 | 40,35 | 47,36 | 46,27 | 42,34 | 28,19 | 32,90 | 51,33 | 24,48 | 36,78 | 54,86 | 24,46 | 55,45 | 18,97 | 30,75 | 40,88 | 41,65 | 46,82 | 48,96 | 49,62 | 3,59 | 46,24 | 45,99 | 30,26 |
| 11 | 46,09 | 4,22 | 49,19 | 61,27 | 18,15 | 31,42 | 37,97 | 43,13 | 51,03 | 46,09 | 43,56 | 28,72 | 37,85 | 49,37 | 25,95 | 38,78 | 52,90 | 26,57 | 60,78 | 20,32 | 29,28 | 42,37 | 45,76 | 49,10 | 48,70 | 51,00 | 3,81 | 48,59 | 47,52 | 28,63 |
| 12 | 50,37 | 2,96 | 49,67 | 61,01 | 23,13 | 32,67 | 44,61 | 43,53 | 43,88 | 50,37 | 46,17 | 29,50 | 36,19 | 51,79 | 26,31 | 34,56 | 57,30 | 27,10 | 59,93 | 22,87 | 33,71 | 44,27 | 47,19 | 45,03 | 49,46 | 51,77 | 2,61 | 49,90 | 49,60 | 29,01 |
| 13 | 49,46 | 1,34 | 50,09 | 63,97 | 18,89 | 33,97 | 45,66 | 38,86 | 46,08 | 49,46 | 45,84 | 31,00 | 41,67 | 51,47 | 26,74 | 38,98 | 54,00 | 26,68 | 59,59 | 19,88 | 32,31 | 40,88 | 40,85 | 46,09 | 49,41 | 50,22 | 1,48 | 45,53 | 46,28 | 31,76 |
| 14 | 52,75 | 7,12 | 51,01 | 56,1 | 17,27 | 35,21 | 43,95 | 41,26 | 48,55 | 52,75 | 42,14 | 27,89 | 38,20 | 56,10 | 30,67 | 37,34 | 54,32 | 29,01 | 63,73 | 20,62 | 33,70 | 46,27 | 43,40 | 46,68 | 51,40 | 52,50 | 4,19 | 53,71 | 44,90 | 29,63 |
| 15 | 47,39 | 1,30 | 51,31 | 60,08 | 23,50 | 31,72 | 38,59 | 43,60 | 42,23 | 47,39 | 46,04 | 26,86 | 38,54 | 52,68 | 24,16 | 38,02 | 58,12 | 24,34 | 55,93 | 20,07 | 32,30 | 41,50 | 44,94 | 42,35 | 48,14 | 48,79 | 0,87 | 46,47 | 49,26 | 29,89 |
| 16 | 48,62 | 1,37 | 56,00 | 59,1 | 25,07 | 34,24 | 43,97 | 47,98 | 46,13 | 48,62 | 50,74 | 25,07 | 39,75 | 59,23 | 23,85 | 40,10 | 59,06 | 24,27 | 63,11 | 22,02 | 29,40 | 47,94 | 44,20 | 46,57 | 50,87 | 54,47 | 0,02 | 50,94 | 49,85 | 27,73 |
| 17 | 46,61 | 1,60 | 49,98 | 58,76 | 21,70 | 25,26 | 41,01 | 43,97 | 40,65 | 46,61 | 44,50 | 21,26 | 38,37 | 53,01 | 20,98 | 32,60 | 44,98 | 20,76 | 57,84 | 19,01 | 27,49 | 32,80 | 33,88 | 39,66 | 45,82 | 47,70 | 0,06 | 43,67 | 39,44 | 22,68 |
| 18 | 50,61 | 3,08 | 52,00 | 59,93 | 19,07 | 34,36 | 44,71 | 44,25 | 50,49 | 50,61 | 43,92 | 21,47 | 40,37 | 55,90 | 26,37 | 34,96 | 56,86 | 26,33 | 60,01 | 20,67 | 35,08 | 43,61 | 44,77 | 54,90 | 49,02 | 55,81 | 3,17 | 50,13 | 46,43 | 23,54 |
| 19 | 46,68 | 0,16 | 58,52 | 62,45 | 31,83 | 31,35 | 44,00 | 49,87 | 43,44 | 46,68 | 56,25 | 22,36 | 36,82 | 63,30 | 22,73 | 39,26 | 59,59 | 26,51 | 69,82 | 25,26 | 29,86 | 51,94 | 45,63 | 43,77 | 52,25 | 56,82 | 1,10 | 57,06 | 50,46 | 23,53 |
| 20 | 47,91 | 2,62 | 56,72 | 58,21 | 21,32 | 30,21 | 42,81 | 48,63 | 45,32 | 47,91 | 51,21 | 27,02 | 33,92 | 60,40 | 27,63 | 38,13 | 57,77 | 27,59 | 64,25 | 23,85 | 26,47 | 42,66 | 49,04 | 45,28 | 48,46 | 60,22 | 2,55 | 49,90 | 51,43 | 26,40 |
| 21 | 50,44 | 4,10 | 55,85 | 59,27 | 25,56 | 33,14 | 46,93 | 48,24 | 46,81 | 50,44 | 53,33 | 28,06 | 40,56 | 61,90 | 26,51 | 37,12 | 58,10 | 25,16 | 59,44 | 25,54 | 31,15 | 43,66 | 43,68 | 46,65 | 50,23 | 56,18 | 1,78 | 50,38 | 49,82 | 26,38 |
| 22 | 46,52 | 2,78 | 48,63 | 61,52 | 16,52 | 29,92 | 43,03 | 40,56 | 45,15 | 46,52 | 40,69 | 21,29 | 35,58 | 52,14 | 26,94 | 37,06 | 47,37 | 25,03 | 58,19 | 12,64 | 27,78 | 34,32 | 35,25 | 43,32 | 46,8 | 48,06 | 1,81 | 46,19 | 38,45 | 21,81 |
| 23 | 47,99 | 3,02 | 54,23 | 56,35 | 23,04 | 34,40 | 44,57 | 48,49 | 47,74 | 47,99 | 49,93 | 27,10 | 33,91 | 61,00 | 26,90 | 34,41 | 55,52 | 24,98 | 61,24 | 24,24 | 36,39 | 45,56 | 40,04 | 46,69 | 48,15 | 52,46 | 2,54 | 48,51 | 47,64 | 27,73 |
| 24 | 52,43 | 5,11 | 55,74 | 65,9 | 30,14 | 36,36 | 48,36 | 53,65 | 46,57 | 52,43 | 54,57 | 28,73 | 39,82 | 63,37 | 25,47 | 41,73 | 61,24 | 22,53 | 60,15 | 22,40 | 33,30 | 42,92 | 50,37 | 44,30 | 52,16 | 53,68 | 2,39 | 52,71 | 50,64 | 25,93 |
| 25 | 49,58 | 0,07 | 43,61 | 56,94 | 15,68 | 28,63 | 44,62 | 36,72 | 39,34 | 49,58 | 37,79 | 26,48 | 38,45 | 49,41 | 26,02 | 36,48 | 45,03 | 21,99 | 56,04 | 14,81 | 27,47 | 41,81 | 36,76 | 42,92 | 49,19 | 40,18 | 1,47 | 48,40 | 37,73 | 25,58 |
| 26 | 48,26 | 0,51 | 50,45 | 56,38 | 23,53 | 27,80 | 43,47 | 43,66 | 39,49 | 48,26 | 43,02 | 23,49 | 39,74 | 53,20 | 23,09 | 33,76 | 53,45 | 21,49 | 52,92 | 24,41 | 27,64 | 39,96 | 42,88 | 38,21 | 46,50 | 53,27 | 3,58 | 46,64 | 44,18 | 22,78 |
| 27 | 48,43 | 3,92 | 48,10 | 54,79 | 23,62 | 33,89 | 39,32 | 44,33 | 43,18 | 48,43 | 45,30 | 25,14 | 37,91 | 54,30 | 24,16 | 34,62 | 54,42 | 23,88 | 49,64 | 18,39 | 33,97 | 36,88 | 41,26 | 43,35 | 48,40 | 45,31 | 4,26 | 46,97 | 41,77 | 24,34 |
| 28 | 49,15 | 1,70 | 54,18 | 58,15 | 21,54 | 28,53 | 41,40 | 51,18 | 43,90 | 49,15 | 48,11 | 24,71 | 39,23 | 60,83 | 25,18 | 38,60 | 63,80 | 24,84 | 62,23 | 25,06 | 24,98 | 42,13 | 52,24 | 42,92 | 50,34 | 56,28 | 0,88 | 49,42 | 54,78 | 24,03 |
| 29 | 50,03 | 4,06 | 58,35 | 63,93 | 25,83 | 29,48 | 50,13 | 50,52 | 42,56 | 50,03 | 61,95 | 25,33 | 38,71 | 65,14 | 21,89 | 32,90 | 63,82 | 22,34 | 63,33 | 23,62 | 29,54 | 44,18 | 49,71 | 44,40 | 48,95 | 58,55 | 4,40 | 49,80 | 53,16 | 23,71 |
| 30 | 50,04 | 2,87 | 51,21 | 58,68 | 27,52 | 35,52 | 44,04 | 45,76 | 46,41 | 50,04 | 49,16 | 25,07 | 39,83 | 58,96 | 24,90 | 37,05 | 60,50 | 25,79 | 52,97 | 24,95 | 34,96 | 40,53 | 46,03 | 46,44 | 51,14 | 50,13 | 3,22 | 47,47 | 49,77 | 28,95 |
