# Supplementary material for: Radiographic evaluation of percutaneous transfacial wiring versus open internal fixation for surgical treatment of unstable zygomatic bone fractures
Source: PLoS One. 2019 Aug 15;14(8):e0220913. doi: 10.1371/journal.pone.0220913 (PMC6695106; doi:10.1371/journal.pone.0220913)
Supplement: S2 Table — For each landmark studied, R corresponds to the right side, L corresponds to the left side, X/Y/Z correlates with the three-dimensional coordinates (X, axial plane; Y, coronal plane; Z, sagittal plane). Or, orbitale landmark; ZFS, zygomaticofrontale suture landmark; Fzf, foramen of the zygomaticofacial nerve; Mp, zygomaxillare point; Zt, zygotemporale inferior point. (DOC) [file pone.0220913.s002.doc]

**S2 Table.**

| ***Patient*** | ***RZFSZ*** | ***RZFSX*** | ***RFZSY*** | ***RZtZ*** | ***RZtY*** | ***RZtX*** | ***RMpZ*** | ***RMpY*** | ***RMpX*** | ***RFzfZ*** | ***RFzfY*** | ***RFzfX*** | ***ROrZ*** | ***ROrY*** | ***ROrX*** | ***LOrZ*** | ***LOrY*** | ***LOrX*** | ***LZtZ*** | ***LZtY*** | ***LZtX*** | ***LMpZ*** | ***LMpY*** | ***LMpX*** | ***LZFSZ*** | ***LZFSY*** | ***LZFSX*** | ***LFzfZ*** | ***LFzfY*** | ***LFzfX*** |
| --- | --- | --- | --- | --- | --- | --- | --- | --- | --- | --- | --- | --- | --- | --- | --- | --- | --- | --- | --- | --- | --- | --- | --- | --- | --- | --- | --- | --- | --- | --- |
| 1 | 46,52 | 0,14 | 56,16 | 60,11 | 27,88 | 27,22 | 36,72 | 44,89 | 38,01 | 46,52 | 49,49 | 21,10 | 34,27 | 51,24 | 21,67 | 37,82 | 62,06 | 23,12 | 60,43 | 30,81 | 28,25 | 41,70 | 53,32 | 41,24 | 49,41 | 60,93 | 0,54 | 48,37 | 57,27 | 25,45 |
| 2 | 47,91 | 3,34 | 52,91 | 56,74 | 16,92 | 28,17 | 42,47 | 38,67 | 45,49 | 47,91 | 45,16 | 32,54 | 35,71 | 51,45 | 28,93 | 37,65 | 50,76 | 28,54 | 56,43 | 12,07 | 29,54 | 43,38 | 37,02 | 46,62 | 48,82 | 48,41 | 3,80 | 46,77 | 44,49 | 32,81 |
| 3 | 50,26 | 1,46 | 57,54 | 63,49 | 25,32 | 30,45 | 42,42 | 42,61 | 45,38 | 50,26 | 48,79 | 28,44 | 38,46 | 51,89 | 23,93 | 38,54 | 62,07 | 22,14 | 61,78 | 30,11 | 25,26 | 44,50 | 49,81 | 43,08 | 51,94 | 59,00 | 0,79 | 50,81 | 55,7 | 24,43 |
| 4 | 52,91 | 5,37 | 55,18 | 59,38 | 20,71 | 35,27 | 46,54 | 49,81 | 50,73 | 52,91 | 47,72 | 30,72 | 43,04 | 62,45 | 27,78 | 39,49 | 63,10 | 24,81 | 59,28 | 24,20 | 37,26 | 47,71 | 50,83 | 49,29 | 51,91 | 52,47 | 4,91 | 53,49 | 51,17 | 29,06 |
| 5 | 50,70 | 5,67 | 51,19 | 58,69 | 22,34 | 30,44 | 43,05 | 45,30 | 41,16 | 50,70 | 45,38 | 22,94 | 38,83 | 52,19 | 25,36 | 40,83 | 53,18 | 25,44 | 57,36 | 23,77 | 31,04 | 40,81 | 45,58 | 41,61 | 51,63 | 52,83 | 5,99 | 51,31 | 47,03 | 23,69 |
| 6 | 49,27 | 3,04 | 53,42 | 62,60 | 15,05 | 33,04 | 45,74 | 47,26 | 49,58 | 49,27 | 49,31 | 23,83 | 37,83 | 59,20 | 22,28 | 35,70 | 60,69 | 22,83 | 63,61 | 21,33 | 30,51 | 45,89 | 49,37 | 47,35 | 47,74 | 54,32 | 2,99 | 50,47 | 51,71 | 23,31 |
| 7 | 48,48 | 1,67 | 52,55 | 63,66 | 21,24 | 30,84 | 40,70 | 44,90 | 45,07 | 48,48 | 41,12 | 21,81 | 31,31 | 52,35 | 29,4 | 36,28 | 56,55 | 21,68 | 57,52 | 22,11 | 29,51 | 39,65 | 50,00 | 39,30 | 46,77 | 52,39 | 0,21 | 49,31 | 46,93 | 19,52 |
| 8 | 49,90 | 0,66 | 61,17 | 65,04 | 24,71 | 27,57 | 43,61 | 43,12 | 44,74 | 49,90 | 51,24 | 24,14 | 38,89 | 59,22 | 25,14 | 37,08 | 64,22 | 26,26 | 59,60 | 24,06 | 31,39 | 43,00 | 47,03 | 46,51 | 48,84 | 58,94 | 2,68 | 48,88 | 55,15 | 27,96 |
| 9 | 48,89 | 1,35 | 51,62 | 59,21 | 21,82 | 31,23 | 46,36 | 46,70 | 43,50 | 48,89 | 47,31 | 23,52 | 41,19 | 57,72 | 24,46 | 37,12 | 60,16 | 25,90 | 56,47 | 21,70 | 34,68 | 48,92 | 47,51 | 47,37 | 48,51 | 52,82 | 2,67 | 54,83 | 47,69 | 28,54 |
| 10 | 47,98 | 3,94 | 53,76 | 57,58 | 22,66 | 31,38 | 39,91 | 45,14 | 47,22 | 47,98 | 49,11 | 30,30 | 32,84 | 57,86 | 28,55 | 32,93 | 53,62 | 24,37 | 62,47 | 22,94 | 28,89 | 42,31 | 40,44 | 44,17 | 48,18 | 54,71 | 0,98 | 46,17 | 46,95 | 27,73 |
| 11 | 47,97 | 1,52 | 47,56 | 55,44 | 19,86 | 29,29 | 39,64 | 34,66 | 40,51 | 47,97 | 39,99 | 22,14 | 33,97 | 47,44 | 23,69 | 35,49 | 52,83 | 24,90 | 55,64 | 22,42 | 30,63 | 43,71 | 38,10 | 41,93 | 47,47 | 50,74 | 2,98 | 47,56 | 44,77 | 23,00 |
| 12 | 48,44 | 0,70 | 57,33 | 59,57 | 21,63 | 31,02 | 47,08 | 47,83 | 43,86 | 48,44 | 50,90 | 20,45 | 36,74 | 65,22 | 23,17 | 32,55 | 62,11 | 25,79 | 59,88 | 23,01 | 33,00 | 45,26 | 44,88 | 44,65 | 47,57 | 57,92 | 0,65 | 50,62 | 51,89 | 21,54 |
| 13 | 48,59 | 1,43 | 45,04 | 55,37 | 14,33 | 30,24 | 41,14 | 32,35 | 45,20 | 48,59 | 42,40 | 30,24 | 38,71 | 48,45 | 26,58 | 38,91 | 43,31 | 27,27 | 58,81 | 11,24 | 31,93 | 42,50 | 28,69 | 45,79 | 49,56 | 41,82 | 2,11 | 46,76 | 37,41 | 30,67 |
| 14 | 48,57 | 2,80 | 49,73 | 61,30 | 12,56 | 39,19 | 41,66 | 45,93 | 44,03 | 48,57 | 43,82 | 23,21 | 38,14 | 56,75 | 25,47 | 38,38 | 61,29 | 26,51 | 63,20 | 23,21 | 35,04 | 44,66 | 47,91 | 45,24 | 49,36 | 54,71 | 2,05 | 54,71 | 49,12 | 23,16 |
| 15 | 47,93 | 4,03 | 48,64 | 57,09 | 14,42 | 32,73 | 42,16 | 43,13 | 53,68 | 47,93 | 43,66 | 29,07 | 38,33 | 54,24 | 26,5 | 35,86 | 58,34 | 26,88 | 59,28 | 17,71 | 33,01 | 41,65 | 44,39 | 52,64 | 48,13 | 50,94 | 3,11 | 49,91 | 45,89 | 30,01 |
| 16 | 52,18 | 1,36 | 51,45 | 60,36 | 25,08 | 32,56 | 48,88 | 47,93 | 44,64 | 52,18 | 51,42 | 29,17 | 38,54 | 56,68 | 23,86 | 38,24 | 56,47 | 25,07 | 55,81 | 20,48 | 34,92 | 38,63 | 40,51 | 46,18 | 54,13 | 48,40 | 2,96 | 46,17 | 45,70 | 31,26 |
| 17 | 49,44 | 2,70 | 58,82 | 61,52 | 26,03 | 31,48 | 44,53 | 51,47 | 43,99 | 49,44 | 55,05 | 25,24 | 37,26 | 61,79 | 22,65 | 37,38 | 63,31 | 22,31 | 58,76 | 26,61 | 29,36 | 41,09 | 47,48 | 40,73 | 50,93 | 59,70 | 2,31 | 46,71 | 53,55 | 24,41 |
| 18 | 49,96 | 0,05 | 61,02 | 62,86 | 26,10 | 25,79 | 48,33 | 49,39 | 42,54 | 49,96 | 53,93 | 20,06 | 36,62 | 65,61 | 22,42 | 33,29 | 63,44 | 22,62 | 61,90 | 30,52 | 25,58 | 42,11 | 44,32 | 40,57 | 48,19 | 62,64 | 0,51 | 46,65 | 54,54 | 24,45 |
| 19 | 48,54 | 6,38 | 57,1 | 57,55 | 29,37 | 35,17 | 41,61 | 49,64 | 45,35 | 48,54 | 51,70 | 28,27 | 37,91 | 59,15 | 27,27 | 33,69 | 56,50 | 25,16 | 53,70 | 23,81 | 28,95 | 35,76 | 45,00 | 41,90 | 48,08 | 56,23 | 4,51 | 46,08 | 47,70 | 26,03 |
| 20 | 49,96 | 3,55 | 52,67 | 59,24 | 26,91 | 37,40 | 44,56 | 47,30 | 44,80 | 49,96 | 48,39 | 29,25 | 37,46 | 57,49 | 26,27 | 33,61 | 54,19 | 26,78 | 53,80 | 23,64 | 37,80 | 39,18 | 40,41 | 44,40 | 49,19 | 51,67 | 5,94 | 48,51 | 45,13 | 30,28 |
| 21 | 51,99 | 1,64 | 59,7 | 62,28 | 24,49 | 29,49 | 43,36 | 48,15 | 46,56 | 51,99 | 54,78 | 27,67 | 39,19 | 63,54 | 27,24 | 30,92 | 56,38 | 24,23 | 62,71 | 24,21 | 27,29 | 35,87 | 40,65 | 42,53 | 50,39 | 60,20 | 1,04 | 46,24 | 50,48 | 26,24 |
| 22 | 47,44 | 0,11 | 54,05 | 59,76 | 22,77 | 30,02 | 42,97 | 48,87 | 43,12 | 47,44 | 47,41 | 22,75 | 35,04 | 59,30 | 23,13 | 34,72 | 58,13 | 24,96 | 55,36 | 22,62 | 31,99 | 40,50 | 47,88 | 46,82 | 47,59 | 53,21 | 1,36 | 48,07 | 47,86 | 27,25 |
| 23 | 50,63 | 5,01 | 50,44 | 59,45 | 19,26 | 38,09 | 41,87 | 47,05 | 44,92 | 50,63 | 43,07 | 29,37 | 37,96 | 55,11 | 28,37 | 35,90 | 57,26 | 28,08 | 61,71 | 20,92 | 34,44 | 46,46 | 46,92 | 43,57 | 50,79 | 53,80 | 1,81 | 51,99 | 47,56 | 28,13 |
| 24 | 48,05 | 2,49 | 53,63 | 54,18 | 32,83 | 34,28 | 40,44 | 46,35 | 42,71 | 48,05 | 50,92 | 26,38 | 32,57 | 55,51 | 23,91 | 31,50 | 52,90 | 22,57 | 57,78 | 20,40 | 28,62 | 38,87 | 39,68 | 39,05 | 45,08 | 52,58 | 0,98 | 43,69 | 46,00 | 22,89 |
| 25 | 51,66 | 0,75 | 51,76 | 61,63 | 18,70 | 34,56 | 45,44 | 46,77 | 47,03 | 51,66 | 45,74 | 26,75 | 40,75 | 57,12 | 25,3 | 35,42 | 50,11 | 26,06 | 68,65 | 21,30 | 32,91 | 47,68 | 42,58 | 50,54 | 52,41 | 50,56 | 0,53 | 55,87 | 46,12 | 24,37 |
| 26 | 46,52 | 0,14 | 56,16 | 60,11 | 27,88 | 27,22 | 36,72 | 44,89 | 38,01 | 46,52 | 49,49 | 21,10 | 34,27 | 51,24 | 21,67 | 37,82 | 62,06 | 23,12 | 60,43 | 30,81 | 28,25 | 41,70 | 53,32 | 41,24 | 49,41 | 60,93 | 0,54 | 48,37 | 57,27 | 25,45 |
| 27 | 48,70 | 0,10 | 46,53 | 59,13 | 18,90 | 29,85 | 44,55 | 40,56 | 40,93 | 48,70 | 44,28 | 25,15 | 39,37 | 51,19 | 24,99 | 36,27 | 50,35 | 28,28 | 63,35 | 17,08 | 29,36 | 45,72 | 36,60 | 40,99 | 50,59 | 44,54 | 1,91 | 52,92 | 38,96 | 25,55 |
| 28 | 49,17 | 0,66 | 55,85 | 60,30 | 28,44 | 28,51 | 48,49 | 50,56 | 38,70 | 49,17 | 53,23 | 20,38 | 38,19 | 62,35 | 21,10 | 33,66 | 64,87 | 21,81 | 57,29 | 33,33 | 27,89 | 45,75 | 53,29 | 38,96 | 47,04 | 59,26 | 3,61 | 48,51 | 56,54 | 21,10 |
| 29 | 44,04 | 2,31 | 53,94 | 53,25 | 28,08 | 25,03 | 40,65 | 47,10 | 36,14 | 44,04 | 47,85 | 17,99 | 34,57 | 58,29 | 21,92 | 27,30 | 53,51 | 23,99 | 49,42 | 27,59 | 24,94 | 30,75 | 40,74 | 38,16 | 42,26 | 55,51 | 1,72 | 37,73 | 45,6 | 21,17 |
| 30 | 47,88 | 0,43 | 55,34 | 60,99 | 23,65 | 32,64 | 43,69 | 44,16 | 43,68 | 47,88 | 47,39 | 26,98 | 36,46 | 59,66 | 25,44 | 35,48 | 57,92 | 26,01 | 61,07 | 18,06 | 31,60 | 38,92 | 40,82 | 45,20 | 49,07 | 54,94 | 3,19 | 47,97 | 45,74 | 29,16 |
