# Supplementary material for: Radiographic evaluation of percutaneous transfacial wiring versus open internal fixation for surgical treatment of unstable zygomatic bone fractures
Source: PLoS One. 2019 Aug 15;14(8):e0220913. doi: 10.1371/journal.pone.0220913 (PMC6695106; doi:10.1371/journal.pone.0220913)
Supplement: S3 Table — For each landmark studied, R corresponds to the right side, L corresponds to the left side, X/Y/Z correlates with the three-dimensional coordinates (X, axial plane; Y, coronal plane; Z, sagittal plane). Or, orbitale landmark; ZFS, zygomaticofrontale suture landmark; Fzf, foramen of the zygomaticofacial nerve; Mp, zygomaxillare point; Zt, zygotemporale inferior point. (DOC) [file pone.0220913.s003.doc]

**S3 Table.**

| ***Patient*** | ***RZFSZ*** | ***RZFSX*** | ***RFZSY*** | ***RZtZ*** | ***RZtY*** | ***RZtX*** | ***RMpZ*** | ***RMpY*** | ***RMpX*** | ***RFzfZ*** | ***RFzfY*** | ***RFzfX*** | ***ROrZ*** | ***ROrY*** | ***ROrX*** | ***LOrZ*** | ***LOrY*** | ***LOrX*** | ***LZtZ*** | ***LZtY*** | ***LZtX*** | ***LMpZ*** | ***LMpY*** | ***LMpX*** | ***LZFSZ*** | ***LZFSY*** | ***LZFSX*** | ***LFzfZ*** | ***LFzfY*** | ***LFzfX*** |
| --- | --- | --- | --- | --- | --- | --- | --- | --- | --- | --- | --- | --- | --- | --- | --- | --- | --- | --- | --- | --- | --- | --- | --- | --- | --- | --- | --- | --- | --- | --- |
| 1 | 51,13 | 1,90 | 54,35 | 64,39 | 19,10 | 30,04 | 44,73 | 42,36 | 48,02 | 51,13 | 45,08 | 24,65 | 41,00 | 55,85 | 26,81 | 36,95 | 57,52 | 29,19 | 62,44 | 19,09 | 32,97 | 42,99 | 43,89 | 50,75 | 49,49 | 54,26 | 3,26 | 51,99 | 43,78 | 26,10 |
| 2 | 47,08 | 2,10 | 52,56 | 58,32 | 23,07 | 32,40 | 45,27 | 45,23 | 45,70 | 47,08 | 47,52 | 22,71 | 37,33 | 59,47 | 27,64 | 34,88 | 61,57 | 26,57 | 58,48 | 27,10 | 29,33 | 43,49 | 48,50 | 45,35 | 46,93 | 56,23 | 1,31 | 50,17 | 49,84 | 19,79 |
| 3 | 50,02 | 1,51 | 53,62 | 60,95 | 27,92 | 31,77 | 47,59 | 54,00 | 41,34 | 50,02 | 55,11 | 25,67 | 40,40 | 60,48 | 21,24 | 37,84 | 58,87 | 22,25 | 54,44 | 32,49 | 32,64 | 44,62 | 49,50 | 40,04 | 48,48 | 53,52 | 1,75 | 47,62 | 50,68 | 23,91 |
| 4 | 49,96 | 1,98 | 55,87 | 59,88 | 23,96 | 31,87 | 44,42 | 48,19 | 46,50 | 49,96 | 53,31 | 30,26 | 34,15 | 62,18 | 28,33 | 34,02 | 58,9 | 28,09 | 60,16 | 20,39 | 30,41 | 44,52 | 45,23 | 45,84 | 49,99 | 53,43 | 0,08 | 47,44 | 53,16 | 30,96 |
| 5 | 53,48 | 0,48 | 50,66 | 62,95 | 21,36 | 35,81 | 42,84 | 47,03 | 47,67 | 53,48 | 46,47 | 27,50 | 39,78 | 54,25 | 26,99 | 36,88 | 48,94 | 24,86 | 61,70 | 15,43 | 30,63 | 41,73 | 38,27 | 44,91 | 50,68 | 49,49 | 1,73 | 50,42 | 42,05 | 23,31 |
| 6 | 48,33 | 0,40 | 61,21 | 56,91 | 31,27 | 32,48 | 44,83 | 55,84 | 45,61 | 48,33 | 55,92 | 26,06 | 41,10 | 66,73 | 27,03 | 39,47 | 64,82 | 27,14 | 59,46 | 27,73 | 31,94 | 41,41 | 54,71 | 46,67 | 48,19 | 61,76 | 0,96 | 49,71 | 55,42 | 28,35 |
| 7 | 42,77 | 1,19 | 45,00 | 53,70 | 18,56 | 29,82 | 44,44 | 45,21 | 43,56 | 42,77 | 45,44 | 24,33 | 34,02 | 56,34 | 21,63 | 34,01 | 50,66 | 20,71 | 52,01 | 16,03 | 28,48 | 40,69 | 39,65 | 41,85 | 43,29 | 44,29 | 0,46 | 43,34 | 42,33 | 24,47 |
| 8 | 49,93 | 0,30 | 47,20 | 67,80 | 19,73 | 32,35 | 51,84 | 38,93 | 46,29 | 49,93 | 41,56 | 24,71 | 43,07 | 51,63 | 27,33 | 41,64 | 53,98 | 24,83 | 65,41 | 23,32 | 30,24 | 48,22 | 42,64 | 44,23 | 50,43 | 51,90 | 0,34 | 51,67 | 47,54 | 24,10 |
| 9 | 53,2 | 2,51 | 54,56 | 64,31 | 19,37 | 33,87 | 44,31 | 46,90 | 52,32 | 53,20 | 47,15 | 27,23 | 38,39 | 59,29 | 28,84 | 39,20 | 58,44 | 28,83 | 61,70 | 20,07 | 31,80 | 44,57 | 47,86 | 51,70 | 54,25 | 56,26 | 2,40 | 53,79 | 47,11 | 25,27 |
| 10 | 47,15 | 1,38 | 51,49 | 56,55 | 21,35 | 31,23 | 42,12 | 43,44 | 46,95 | 47,15 | 45,35 | 28,01 | 37,38 | 55,77 | 24,46 | 37,78 | 56,60 | 24,17 | 54,66 | 19,50 | 30,81 | 41,81 | 41,33 | 45,65 | 48,36 | 50,47 | 2,19 | 47,04 | 45,51 | 30,33 |
| 11 | 46,08 | 4,56 | 51,25 | 63,18 | 20,26 | 32,79 | 42,24 | 47,16 | 52,57 | 46,08 | 48,11 | 33,83 | 35,30 | 52,66 | 28,92 | 37,18 | 54,99 | 27,15 | 60,79 | 19,42 | 29,04 | 42,55 | 46,82 | 49,85 | 48,39 | 52,62 | 2,69 | 49,39 | 47,60 | 30,94 |
| 12 | 51,94 | 4,44 | 52,87 | 61,41 | 23,83 | 35,35 | 45,57 | 48,58 | 47,18 | 51,94 | 49,29 | 32,11 | 32,82 | 57,55 | 30,38 | 36,48 | 55,15 | 26,49 | 59,09 | 21,11 | 32,44 | 45,51 | 45,39 | 43,71 | 49,28 | 51,28 | 1,16 | 50,40 | 49,21 | 33,19 |
| 13 | 49,55 | 3,31 | 51,39 | 60,60 | 15,30 | 34,11 | 46,31 | 39,02 | 46,86 | 49,55 | 44,35 | 33,30 | 43,09 | 53,02 | 29,85 | 38,85 | 51,36 | 29,97 | 59,04 | 17,26 | 33,77 | 40,86 | 38,40 | 47,52 | 48,79 | 49,97 | 4,49 | 44,45 | 44,53 | 33,13 |
| 14 | 54,11 | 8,23 | 53,64 | 61,76 | 20,04 | 36,29 | 46,31 | 45,37 | 49,59 | 54,11 | 45,23 | 28,42 | 41,18 | 56,39 | 31,97 | 39,46 | 56,16 | 30,87 | 62,74 | 22,75 | 35,62 | 45,91 | 47,04 | 48,44 | 51,98 | 54,23 | 6,57 | 53,37 | 47,41 | 31,10 |
| 15 | 49,47 | 3,75 | 52,98 | 61,44 | 26,39 | 31,81 | 43,38 | 50,46 | 42,26 | 49,47 | 51,07 | 26,50 | 37,45 | 61,34 | 22,79 | 33,30 | 61,16 | 23,73 | 55,17 | 20,79 | 33,02 | 38,79 | 46,19 | 41,20 | 47,56 | 49,31 | 1,96 | 45,66 | 51,84 | 29,7 |
| 16 | 47,39 | 2,20 | 50,57 | 65,27 | 14,54 | 30,69 | 47,91 | 49,68 | 46,32 | 47,39 | 51,50 | 24,46 | 34,85 | 63,08 | 24,58 | 33,45 | 60,93 | 24,98 | 63,49 | 13,75 | 28,59 | 46,77 | 45,05 | 47,72 | 49,3 | 55,51 | 0,11 | 50,17 | 49,41 | 29,28 |
| 17 | 46,44 | 1,01 | 50,35 | 58,13 | 22,74 | 25,55 | 39,68 | 45,38 | 40,78 | 46,44 | 43,44 | 23,92 | 38,15 | 53,47 | 21,35 | 36,81 | 51,85 | 22,23 | 58,91 | 17,81 | 25,71 | 39,28 | 41,16 | 40,57 | 46,64 | 48,74 | 0,20 | 48,73 | 41,76 | 23,59 |
| 18 | 50,06 | 1,87 | 55,85 | 61,30 | 20,83 | 34,79 | 45,70 | 46,63 | 50,41 | 50,06 | 53,39 | 33,59 | 39,36 | 58,17 | 27,18 | 35,76 | 58,85 | 27,47 | 60,78 | 21,29 | 35,69 | 45,45 | 48,67 | 53,66 | 48,15 | 56,14 | 1,64 | 47,84 | 52,40 | 32,87 |
| 19 | 47,89 | 1,35 | 56,02 | 61,78 | 28,57 | 33,41 | 43,78 | 50,23 | 44,18 | 47,89 | 55,76 | 24,57 | 33,92 | 64,95 | 24,49 | 37,15 | 59,74 | 22,84 | 60,72 | 26,13 | 24,21 | 46,73 | 53,50 | 44,41 | 47,57 | 56,87 | 2,57 | 52,25 | 52,47 | 20,08 |
| 20 | 48,14 | 2,63 | 57,84 | 59,49 | 20,45 | 30,12 | 43,25 | 49,79 | 45,21 | 48,14 | 51,78 | 27,35 | 37,35 | 57,25 | 26,58 | 37,48 | 59,26 | 26,51 | 63,84 | 26,26 | 27,52 | 44,24 | 54,83 | 44,89 | 47,98 | 60,63 | 1,49 | 49,93 | 56,02 | 28,79 |
| 21 | 51,11 | 3,88 | 55,91 | 60,48 | 25,79 | 33,76 | 45,85 | 48,23 | 47,32 | 51,11 | 54,30 | 28,73 | 37,99 | 64,03 | 27,63 | 38,12 | 60,28 | 26,51 | 59,73 | 25,52 | 31,86 | 44,90 | 47,76 | 47,98 | 50,22 | 55,29 | 2,48 | 51,30 | 51,76 | 27,42 |
| 22 | 45,56 | 1,76 | 48,41 | 59,75 | 17,27 | 29,61 | 42,46 | 40,40 | 44,15 | 45,56 | 41,75 | 29,10 | 37,06 | 51,37 | 25,01 | 36,07 | 52,44 | 25,42 | 60,15 | 13,64 | 26,96 | 42,16 | 42,00 | 45,44 | 47,65 | 49,54 | 1,13 | 48,66 | 41,21 | 29,94 |
| 23 | 48,09 | 1,19 | 57,68 | 56,92 | 28,47 | 33,700 | 46,40 | 51,35 | 44,38 | 48,09 | 53,94 | 23,75 | 39,80 | 63,04 | 23,76 | 37,43 | 64,12 | 24,30 | 60,78 | 27,60 | 35,50 | 47,62 | 48,77 | 45,84 | 49,41 | 56,83 | 2,48 | 51,03 | 53,87 | 25,63 |
| 24 | 53,77 | 5,05 | 56,92 | 66,93 | 26,89 | 33,64 | 47,51 | 55,69 | 46,36 | 53,77 | 55,36 | 27,95 | 45,52 | 62,75 | 25,28 | 44,81 | 64,52 | 23,49 | 64,55 | 27,28 | 35,62 | 47,03 | 54,57 | 45,00 | 53,23 | 55,49 | 2,81 | 55,04 | 54,78 | 26,04 |
| 25 | 50,14 | 0,67 | 42,71 | 57,92 | 14,85 | 28,05 | 44,09 | 38,29 | 39,88 | 50,14 | 38,30 | 25,68 | 40,49 | 47,67 | 25,48 | 38,45 | 51,73 | 24,97 | 54,90 | 16,16 | 29,45 | 45,61 | 40,33 | 39,47 | 49,87 | 40,61 | 0,77 | 51,67 | 38,81 | 27,40 |
| 26 | 48,43 | 0,41 | 51,07 | 56,75 | 23,60 | 27,37 | 45,15 | 44,80 | 39,24 | 48,43 | 43,42 | 24,50 | 40,75 | 52,46 | 23,64 | 37,87 | 54,15 | 23,86 | 55,90 | 24,55 | 27,80 | 45,57 | 44,26 | 39,15 | 46,81 | 51,92 | 2,22 | 50,21 | 42,65 | 24,21 |
| 27 | 48,07 | 3,39 | 46,04 | 55,40 | 21,80 | 33,16 | 39,47 | 44,25 | 42,13 | 48,07 | 44,60 | 24,27 | 37,13 | 54,56 | 24,25 | 35,72 | 54,64 | 24,68 | 54,88 | 21,44 | 35,63 | 39,45 | 43,43 | 44,04 | 46,72 | 44,84 | 3,01 | 48,30 | 43,21 | 24,76 |
| 28 | 50,34 | 1,56 | 55,11 | 59,03 | 21,83 | 27,10 | 41,47 | 49,92 | 43,30 | 50,34 | 48,18 | 24,07 | 40,72 | 59,04 | 23,83 | 40,45 | 62,33 | 24,47 | 62,11 | 25,19 | 23,86 | 41,25 | 51,36 | 42,82 | 50,86 | 56,59 | 0,74 | 50,38 | 52,68 | 22,26 |
| 29 | 50,72 | 2,27 | 59,07 | 62,56 | 25,90 | 32,43 | 47,32 | 50,29 | 45,76 | 50,72 | 53,75 | 21,24 | 39,66 | 66,14 | 24,10 | 35,38 | 64,96 | 23,26 | 62,73 | 26,78 | 31,04 | 47,74 | 50,50 | 44,81 | 49,57 | 58,40 | 3,03 | 51,95 | 53,59 | 23,78 |
| 30 | 49,74 | 2,13 | 50,51 | 58,80 | 27,04 | 35,28 | 44,11 | 46,26 | 45,35 | 49,74 | 49,10 | 24,68 | 41,93 | 56,69 | 24,21 | 38,90 | 60,30 | 25,53 | 54,95 | 23,88 | 34,02 | 42,86 | 44,91 | 45,03 | 50,43 | 49,29 | 2,14 | 48,41 | 48,93 | 27,57 |
