# Supplementary material for: Radiographic evaluation of percutaneous transfacial wiring versus open internal fixation for surgical treatment of unstable zygomatic bone fractures
Source: PLoS One. 2019 Aug 15;14(8):e0220913. doi: 10.1371/journal.pone.0220913 (PMC6695106; doi:10.1371/journal.pone.0220913)
Supplement: S4 Table — For each landmark studied, R corresponds to the right side, L corresponds to the left side, X/Y/Z correlates with the three-dimensional coordinates (X, axial plane; Y, coronal plane; Z, sagittal plane). Or, orbitale landmark; ZFS, zygomaticofrontale suture landmark; Fzf, foramen of the zygomaticofacial nerve; Mp, zygomaxillare point; Zt, zygotemporale inferior point. (DOC) [file pone.0220913.s004.doc]

**S4 Table.**

| ***Patient*** | ***RZFSZ*** | ***RZFSX*** | ***RFZSY*** | ***RZtZ*** | ***RZtY*** | ***RZtX*** | ***RMpZ*** | ***RMpY*** | ***RMpX*** | ***RFzfZ*** | ***RFzfY*** | ***RFzfX*** | ***ROrZ*** | ***ROrY*** | ***ROrX*** | ***LOrZ*** | ***LOrY*** | ***LOrX*** | ***LZtZ*** | ***LZtY*** | ***LZtX*** | ***LMpZ*** | ***LMpY*** | ***LMpX*** | ***LZFSZ*** | ***LZFSY*** | ***LZFSX*** | ***LFzfZ*** | ***LFzfY*** | ***LFzfX*** |
| --- | --- | --- | --- | --- | --- | --- | --- | --- | --- | --- | --- | --- | --- | --- | --- | --- | --- | --- | --- | --- | --- | --- | --- | --- | --- | --- | --- | --- | --- | --- |
| 1 | 48,82 | 0,91 | 56,19 | 61,33 | 27,73 | 27,83 | 42,55 | 49,18 | 40,33 | 48,82 | 50,23 | 22,70 | 36,94 | 56,45 | 25,46 | 37,10 | 59,11 | 25,06 | 59,58 | 28,65 | 30,00 | 41,35 | 51,40 | 42,82 | 48,85 | 58,25 | 1,36 | 47,62 | 54,01 | 28,06 |
| 2 | 47,85 | 2,04 | 53,92 | 56,95 | 19,26 | 27,91 | 43,52 | 41,76 | 45,22 | 47,85 | 47,23 | 31,21 | 37,02 | 52,94 | 27,73 | 38,21 | 51,88 | 29,56 | 55,33 | 12,97 | 31,41 | 43,46 | 37,90 | 48,01 | 48,88 | 47,94 | 5,34 | 46,27 | 45,55 | 34,00 |
| 3 | 51,04 | 3,05 | 58,00 | 63,71 | 26,38 | 30,22 | 45,91 | 51,01 | 49,09 | 51,04 | 54,08 | 28,69 | 38,72 | 61,74 | 27,76 | 37,8 | 61,78 | 22,28 | 60,67 | 27,38 | 24,95 | 43,94 | 49,86 | 42,82 | 50,45 | 58,42 | 2,20 | 50,38 | 54,53 | 24,30 |
| 4 | 52,10 | 4,57 | 53,16 | 62,42 | 22,26 | 34,73 | 48,83 | 52,42 | 49,26 | 52,10 | 49,53 | 28,72 | 40,08 | 62,48 | 25,10 | 39,98 | 63,74 | 24,19 | 60,10 | 24,71 | 36,80 | 48,42 | 51,36 | 48,91 | 52,25 | 52,81 | 4,86 | 53,46 | 52,00 | 27,70 |
| 5 | 50,80 | 4,25 | 51,17 | 59,42 | 21,31 | 29,15 | 41,9 | 43,69 | 40,54 | 50,8 | 44,67 | 21,83 | 39,86 | 51,63 | 24,79 | 39,14 | 52,18 | 26,51 | 56,65 | 21,04 | 31,76 | 41,25 | 43,91 | 41,92 | 50,49 | 51,39 | 6,90 | 51,50 | 44,47 | 23,57 |
| 6 | 49,26 | 3,12 | 54,15 | 64,42 | 18,96 | 32,96 | 44,89 | 49,49 | 49,53 | 49,26 | 50,94 | 24,17 | 36,24 | 62,03 | 22,29 | 35,7 | 61,59 | 22,23 | 63,51 | 23,80 | 30,41 | 45,26 | 50,59 | 46,79 | 48,35 | 56,46 | 0,94 | 51,17 | 51,81 | 23,41 |
| 7 | 47,46 | 1,47 | 51,68 | 61,45 | 22,51 | 30,84 | 36,13 | 47,40 | 43,03 | 47,46 | 42,60 | 19,50 | 37,00 | 53,60 | 24,14 | 34,38 | 57,77 | 24,80 | 58,23 | 22,34 | 32,07 | 35,57 | 51,08 | 44,01 | 47,68 | 54,70 | 1,79 | 49,13 | 47,63 | 22,46 |
| 8 | 50,67 | 0,48 | 63,03 | 63,77 | 33,26 | 29,19 | 45,92 | 48,12 | 44,68 | 50,67 | 54,99 | 23,09 | 42,72 | 62,59 | 24,62 | 38,72 | 61,24 | 26,39 | 58,47 | 23,47 | 30,22 | 44,39 | 45,26 | 45,26 | 49,08 | 59,94 | 3,91 | 48,32 | 55,39 | 27,18 |
| 9 | 49,19 | 1,57 | 52,76 | 61,27 | 21,66 | 31,36 | 47,03 | 44,88 | 44,06 | 49,19 | 47,52 | 24,34 | 41,43 | 55,99 | 25,03 | 37,42 | 58,42 | 27,23 | 58,13 | 18,91 | 37,13 | 44,45 | 43,21 | 47,40 | 49,31 | 51,91 | 2,69 | 52,16 | 43,31 | 24,56 |
| 10 | 48,07 | 3,68 | 52,92 | 57,77 | 22,45 | 30,91 | 40,08 | 45,42 | 47,14 | 48,07 | 49,31 | 30,41 | 33,54 | 56,37 | 28,08 | 35,28 | 56,51 | 26,62 | 61,95 | 24,55 | 30,83 | 46,46 | 44,89 | 46,99 | 49,15 | 54,83 | 2,82 | 49,56 | 50,54 | 29,86 |
| 11 | 47,81 | 0,16 | 47,16 | 56,85 | 24,00 | 29,78 | 44,33 | 40,88 | 40,40 | 47,81 | 42,52 | 19,43 | 34,07 | 53,55 | 23,17 | 34,53 | 53,26 | 22,93 | 55,69 | 24,13 | 30,57 | 44,04 | 41,08 | 41,02 | 48,15 | 50,40 | 1,54 | 48,16 | 45,39 | 22,02 |
| 12 | 49,48 | 0,34 | 56,98 | 59,01 | 22,48 | 30,46 | 46,92 | 47,16 | 42,81 | 49,48 | 52,72 | 23,96 | 36,44 | 65,40 | 23,23 | 39,57 | 63,71 | 23,92 | 59,37 | 25,46 | 33,23 | 46,17 | 46,55 | 44,44 | 48,34 | 58,39 | 1,46 | 53,00 | 49,54 | 21,90 |
| 13 | 48,99 | 1,59 | 45,80 | 55,97 | 14,72 | 30,91 | 44,34 | 32,70 | 43,51 | 48,99 | 42,53 | 30,13 | 38,25 | 47,89 | 26,47 | 38,22 | 43,75 | 27,51 | 58,41 | 11,70 | 32,72 | 44,32 | 28,67 | 45,48 | 49,51 | 41,57 | 1,91 | 48,07 | 36,89 | 30,68 |
| 14 | 49,93 | 2,89 | 50,96 | 61,80 | 15,56 | 38,68 | 43,92 | 47,64 | 43,63 | 49,93 | 45,14 | 23,42 | 37,65 | 56,85 | 23,83 | 34,86 | 59,15 | 25,97 | 60,62 | 21,65 | 34,81 | 43,70 | 44,20 | 46,44 | 48,85 | 54,83 | 2,64 | 51,66 | 48,99 | 23,42 |
| 15 | 48,35 | 2,91 | 47,52 | 57,48 | 12,45 | 30,47 | 43,17 | 39,62 | 52,72 | 48,35 | 42,02 | 28,01 | 38,04 | 53,33 | 26,38 | 35,30 | 55,50 | 27,56 | 58,53 | 15,66 | 33,31 | 42,16 | 41,50 | 52,71 | 48,19 | 49,33 | 4,35 | 49,32 | 43,26 | 30,42 |
| 16 | 51,05 | 1,42 | 50,49 | 60,54 | 23,86 | 33,20 | 47,10 | 46,93 | 46,66 | 51,05 | 50,55 | 29,87 | 37,46 | 56,73 | 24,58 | 40,48 | 52,32 | 25,61 | 62,00 | 20,15 | 36,03 | 45,50 | 43,89 | 48,57 | 50,81 | 46,13 | 3,87 | 52,00 | 44,53 | 30,91 |
| 17 | 50,09 | 2,97 | 56,49 | 62,05 | 26,21 | 32,77 | 43,61 | 50,32 | 44,32 | 50,09 | 53,32 | 25,49 | 39,87 | 59,34 | 22,21 | 37,30 | 62,02 | 21,95 | 60,63 | 28,11 | 31,62 | 43,15 | 52,47 | 43,38 | 50,00 | 58,57 | 1,97 | 48,48 | 56,29 | 25,04 |
| 18 | 50,05 | 2,25 | 60,88 | 62,48 | 26,24 | 25,00 | 50,09 | 46,92 | 41,69 | 50,05 | 53,29 | 19,66 | 40,50 | 63,71 | 21,62 | 35,71 | 65,46 | 25,49 | 60,95 | 29,03 | 28,08 | 49,59 | 46,07 | 43,21 | 49,05 | 62,56 | 0,48 | 49,81 | 55,97 | 27,13 |
| 19 | 48,23 | 6,41 | 56,66 | 57,97 | 28,38 | 35,60 | 42,53 | 49,51 | 45,13 | 48,23 | 51,54 | 29,40 | 36,50 | 57,54 | 26,86 | 32,21 | 57,20 | 24,01 | 57,65 | 27,29 | 31,78 | 41,00 | 47,13 | 41,50 | 48,10 | 54,17 | 1,94 | 47,44 | 49,79 | 26,27 |
| 20 | 49,74 | 2,66 | 53,05 | 59,24 | 27,55 | 37,12 | 45,16 | 46,71 | 43,74 | 49,74 | 49,39 | 27,38 | 39,46 | 57,15 | 24,88 | 37,04 | 57,79 | 26,72 | 56,53 | 24,67 | 37,98 | 43,62 | 43,82 | 45,26 | 50,79 | 52,35 | 4,89 | 52,24 | 47,98 | 29,28 |
| 21 | 51,56 | 3,00 | 59,05 | 62,85 | 23,41 | 27,24 | 43,28 | 48,31 | 45,84 | 51,56 | 54,20 | 26,48 | 41,11 | 62,20 | 25,55 | 35,28 | 62,15 | 28,99 | 62,64 | 23,97 | 27,95 | 39,25 | 48,43 | 49,10 | 51,13 | 57,63 | 1,73 | 51,61 | 52,01 | 28,31 |
| 22 | 48,17 | 1,16 | 52,73 | 60,31 | 21,72 | 30,52 | 42,27 | 48,13 | 43,84 | 48,17 | 46,48 | 24,30 | 34,45 | 58,25 | 24,22 | 33,91 | 57,50 | 23,10 | 58,66 | 22,21 | 30,58 | 42,75 | 47,11 | 44,78 | 46,53 | 48,32 | 0,00 | 49,10 | 47,44 | 25,51 |
| 23 | 50,53 | 5,05 | 48,46 | 58,81 | 18,16 | 37,29 | 42,69 | 46,36 | 43,94 | 50,53 | 41,61 | 24,90 | 36,97 | 57,69 | 28,38 | 38,17 | 56,97 | 27,01 | 62,09 | 19,66 | 33,49 | 45,72 | 48,80 | 42,56 | 51,48 | 51,88 | 2,84 | 54,68 | 44,01 | 22,79 |
| 24 | 48,66 | 3,72 | 53,27 | 59,79 | 23,16 | 33,09 | 41,83 | 47,91 | 42,85 | 48,66 | 50,77 | 26,20 | 37,63 | 55,24 | 24,37 | 34,49 | 54,42 | 22,20 | 60,81 | 22,70 | 29,3 | 41,69 | 47,60 | 40,24 | 48,39 | 52,19 | 0,31 | 47,62 | 48,27 | 22,82 |
| 25 | 51,81 | 1,78 | 51,16 | 61,74 | 17,16 | 35,7 | 44,48 | 46,68 | 48,25 | 51,81 | 45,68 | 27,71 | 42,21 | 54,23 | 25,63 | 38,34 | 60,87 | 24,53 | 64,96 | 20,63 | 34,09 | 45,29 | 49,61 | 47,50 | 52,04 | 52,29 | 0,10 | 56,78 | 48,06 | 23,02 |
| 26 | 48,82 | 0,91 | 56,19 | 61,33 | 27,73 | 27,83 | 42,55 | 49,18 | 40,33 | 48,82 | 50,23 | 22,70 | 36,94 | 56,45 | 25,46 | 37,10 | 59,11 | 25,06 | 59,58 | 28,65 | 30,00 | 41,35 | 51,40 | 42,82 | 48,85 | 58,25 | 1,36 | 47,62 | 54,01 | 28,06 |
| 27 | 47,52 | 0,28 | 46,76 | 58,89 | 18,26 | 29,68 | 45,37 | 38,83 | 40,68 | 47,52 | 44,76 | 24,85 | 38,31 | 53,24 | 25,40 | 35,76 | 51,16 | 26,87 | 60,34 | 18,05 | 31,79 | 45,36 | 39,20 | 41,11 | 50,06 | 46,26 | 1,36 | 52,76 | 42,03 | 24,90 |
| 28 | 48,77 | 2,63 | 54,93 | 57,43 | 29,06 | 31,79 | 45,00 | 48,89 | 43,04 | 48,77 | 52,24 | 24,36 | 36,30 | 60,73 | 25,02 | 35,10 | 60,78 | 23,36 | 58,66 | 30,09 | 28,41 | 44,91 | 47,66 | 40,45 | 47,90 | 57,36 | 1,57 | 50,06 | 53,80 | 22,96 |
| 29 | 43,59 | 1,01 | 53,20 | 53,82 | 27,79 | 26,15 | 40,69 | 47,11 | 37,46 | 43,59 | 48,03 | 20,02 | 35,23 | 57,51 | 22,78 | 33,55 | 60,38 | 26,02 | 52,52 | 29,11 | 26,56 | 37,55 | 46,76 | 39,88 | 42,62 | 56,08 | 1,37 | 43,77 | 48,38 | 23,25 |
| 30 | 47,81 | 1,31 | 54,72 | 60,82 | 22,56 | 31,71 | 43,85 | 44,15 | 43,12 | 47,81 | 46,67 | 27,55 | 35,45 | 60,33 | 26,18 | 34,45 | 59,66 | 26,43 | 59,94 | 21,47 | 31,80 | 41,79 | 41,79 | 44,61 | 48,09 | 54,62 | 3,86 | 49,50 | 47,27 | 28,30 |
